# Abo1 is required for the H3K9me2 to H3K9me3 transition in heterochromatin

**DOI:** 10.1038/s41598-020-63209-y

**Published:** 2020-04-08

**Authors:** Wenbo Dong, Eriko Oya, Yasaman Zahedi, Punit Prasad, J. Peter Svensson, Andreas Lennartsson, Karl Ekwall, Mickaël Durand-Dubief

**Affiliations:** 1 Department of Biosciences and Nutrition, Karolinska Institutet NEO, 141 83 Huddinge, Sweden; 2 Institute of Life Sciences, NALCO Square, Bhubaneswar, 751023 Odhisa India

**Keywords:** Centromeres, Centromeres, Telomeres, Telomeres

## Abstract

Heterochromatin regulation is critical for genomic stability. Different H3K9 methylation states have been discovered, with distinct roles in heterochromatin formation and silencing. However, how the transition from H3K9me2 to H3K9me3 is controlled is still unclear. Here, we investigate the role of the conserved bromodomain AAA-ATPase, Abo1, involved in maintaining global nucleosome organisation in fission yeast. We identified several key factors involved in heterochromatin silencing that interact genetically with Abo1: histone deacetylase Clr3, H3K9 methyltransferase Clr4, and HP1 homolog Swi6. Cells lacking Abo1 cultivated at 30 °C exhibit an imbalance of H3K9me2 and H3K9me3 in heterochromatin. In *abo1*∆ cells, the centromeric constitutive heterochromatin has increased H3K9me2 but decreased H3K9me3 levels compared to wild-type. In contrast, facultative heterochromatin regions exhibit reduced H3K9me2 and H3K9me3 levels in *abo1*∆. Genome-wide analysis showed that *abo1*∆ cells have silencing defects in both the centromeres and subtelomeres, but not in a subset of heterochromatin islands in our condition. Thus, our work uncovers a role of Abo1 in stabilising directly or indirectly Clr4 recruitment to allow the H3K9me2 to H3K9me3 transition in heterochromatin.

## Introduction

In eukaryotic cells, the regions of the chromatin that contain active genes are termed euchromatin, and these regions condense in mitosis to allow for chromosome segregation and decondense in interphase to allow for gene transcription^1^. The chromatin regions that remain condensed throughout the cell cycle are defined as heterochromatin regions and are transcriptionally repressed^2,3^. In general, heterochromatin can be defined molecularly by the modification of histone H3, specifically the absence of methylation on lysine 4 (H3K4me) and presence of methylation on lysine 9 (H3K9me)^4–6^. A conserved group of heterochromatin proteins, the heterochromatin protein 1 (HP1) family, specifically recognise heterochromatin regions by binding to H3K9me. Both di- and tri-methylation (H3K9me2/3) enable the binding of HP1 proteins to silence heterochromatin^7,8^. Heterochromatin regions can be further classified into constitutive and facultative heterochromatin^9,10^. Fission yeast, *Schizosaccharomyces pombe*, is an excellent model organism for chromatin studies because its heterochromatin organisation is similar to human cells^11,12^. Examples of constitutive heterochromatin regions in *S. pombe* are the pericentromeric regions that contain repetitive DNA sequences^13^. These regions are regulated by RNAi-dependent silencing machinery that involves protein Dcr1 and Ago1^14,15^. In contrast, the silencing of facultative heterochromatin, such as subtelomeric regions and heterochromatin islands, is mediated by RNAi-independent machinery that can be dynamically modulated to express genes required for the cell cycle or adaptation to the cell environment^16,17^. A subset of facultative heterochromatin islands, known as “determinant of selective removal” (DSR) islands, contain meiotic genes^18^. These islands are silenced through a specific RNA elimination process, together with Clr4, when the cells are not in meiosis^19^.

Distinct roles have been observed for H3K9me2 and H3K9me3 in heterochromatin silencing. In the nematode *Caenorhabditis elegans*, H3K9 is methylated sequentially and H3K9me2 and H3K9me3 are present in distinct localised chromatin domains^20,21^. Jih *et al*. recently reported that the transition from H3K9me2 to H3K9me3 can be blocked by introducing point mutations in the SET and chromodomain of *S. pombe* Clr4 (I418P, F449Y, and W31G)^22^. These three Clr4 mutants exhibit increased H3K9me2 and reduced H3K9me3 levels in pericentromeric regions, which allows the transcription of *dg*-*dh* repeats^22^. However, the two Clr4 SET domain mutants (I418P and F449Y) block the transition to H3K9me3 in the pericentromeric region by inhibiting the methylation capacity of the enzyme, whereas the chromodomain mutant Clr4 (W31G) reduces the transition to H3K9me3 by blocking recognition and binding to methylated H3K9^22,23^.

In *S. pombe*, H3K9me2 regions allow transcription, whereas H3K9me3 regions are transcriptionally silent^22^. The Clr4 enzyme is comprised of the chromodomain and SET domains and binds to methylated H3K9^24^. The HP1 homologue Swi6 also binds H3K9me and acts together with the histone deacetylase Clr3 to prevent histone turnover in heterochromatin regions, repressing transcription^25^. Clr4 is responsible for both H3K9me2 and H3K9me3 in fission yeast, but the determinants and auxiliary proteins leading to the transition from H3K9me2 to H3K9me3 remain unknown.

The bromodomain is an extensive evolutionarily conserved protein domain that recognises acetylated lysine residues and plays multiple roles in transcriptional activation^26^. The human bromodomain-containing proteins, ATAD2 and ATAD2B, belong to the AAA (ATPases Associated with diverse cellular Activities) ATPase family. Proteins belonging to this family hydrolyse ATP to mediate conformational changes in substrate proteins, resulting in diverse cellular processes^27,28^. ATAD2 plays a key role in cell proliferation and is upregulated as a potential oncogene in cervical, colorectal, breast, and lung cancers^29–32^. In the budding yeast *Saccharomyces cerevisiae*, the ATAD2 homologue Yta7 binds to histones and participates in the heterochromatin boundary function in the HMR region, where it counteracts the spread of silencing into the neighbouring euchromatin^33,34^. This ScYta7 boundary function is possibly related to the regulation of nucleosome density through eviction of H3-H4 tetramers from the chromatin^35^.

Two AAA-ATPase bromodomain family members have been identified in *S. pombe*, Abo1 and Abo2^36^. A recent study showed *in vitro* that Abo1 structure works as a hexamer using Cryo-EM analysis and is able to load H3-H4 onto DNA in a ATP-hydrolysis dependent manner^37^. *In vivo*, cells lacking Abo1 exhibit a global reduction in nucleosome levels, nucleosome redistribution, silencing defects at centromeric heterochromatin regions, and a chromosome segregation phenotype. Abo1 plays a role in centromere formation, as Abo1 is localised at pericentromeric heterochromatin and *abo1*∆ mutant cells present with silencing and defects in chromosome segregation ^38^Intriguingly, pericentromeric heterochromatin in *abo1*∆ has a slight increase in H3K9me2 enrichment, with clear defects in silencing^38^. However, the mechanism underlying Abo1 function is still unknown.

Here, our synthetic genetic screening of Abo1-interacting genes in *S. pombe* revealed several heterochromatin factors, including the histone deacetylase Clr3, H3K9 methyltransferase Clr4, and HP1 homologue Swi6. We found that cells lacking *abo1* exhibit a strong imbalance in H3K9me2 and H3K9me3 levels within different heterochromatin regions, resulting in transcriptional deregulation. We show that Abo1 is involved in the heterochromatin assembly process by stabilising Clr4 binding and mediating the transition between H3K9me2 and H3K9me3.

## Results

### Abo1 interacts genetically with heterochromatin factors Clr3, Clr4, and Swi6

To explore the function of Abo1 in chromatin structure and function, we conducted a focused genetic screen to identify partners of Abo1. We performed a Synthetic Genetic Array (SGA) assay^39^ in which *S. pombe* strains harbouring deletions of genes involved in chromatin regulation were crossed with a strain carrying an *abo1* deletion. Among 711 investigated genes, the strongest negative genetic interactions were recorded with genes encoding proteins involved in heterochromatin assembly: histone deacetylase Clr3, H3K9 methyltransferase Clr4, and chromodomain HP1 homologue Swi6 (Supplementary Table S1).

**Figure 1:**
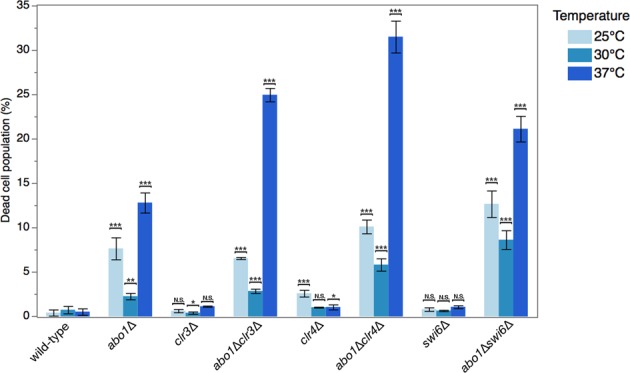
Abo1 interacts genetically with heterochromatin factors Clr3, Clr4, and Swi6. A histogram showing the results of flow cytometry analyses of the population of dead cells of single *clr3∆*, *clr4∆*, and *swi6∆* mutants and their corresponding double mutants combined with *abo1* deletion at normal culture temperature (30 °C) or under cold stress (25 °C) and heat stress (37 °C) conditions for 4 hours. The following strains were used: Hu2185, Hu2318, Hu3022, Hu3026, Hu3023, Hu3027, Hu3024, and Hu3028. The percentage of dead cells at different temperatures for the different deleted mutants. Error bars indicate standard error of the mean (n = 3). *p < 0.1, **p < 0.05, ***p < 0.01, two-tailed unpaired t-test. N.S., not significant (i.e., p > 0.1).

Other bromodomain family members in human cells are implicated in the modulation of gene expression in heterochromatin during temperature stress^40^. Therefore, we also analysed the cell viability of wild-type and *abo1* single/double-mutant cells during temperature stress. At the standard growth temperature (30 °C), the population of dead cells was increased in the *abo1*∆ culture compared to wild-type. A synergistic increase in cell death was observed for the double mutants of *abo1*∆ combined with *clr4*∆ and *swi6*∆ (proportion of dead cells for triplicate culture in WT: 0.76 ± 0.41%*; abo1∆*: 2.28 ± 0.36%; *clr4*∆: 1.04 ± 0.05%; *abo1∆clr4*∆: 5.85 ± 0.69%; *swi6*∆: 0.67 ± 0.07%; *abo1∆swi6*∆: 8.65 ± 1.06%). No additive effects were detected with *abo1∆* and *clr3∆* (*clr3*∆: 0.43% ± 0.13; *abo1∆clr3*∆: 2.88% ± 0.24; Fig. 1). Growth experiments under cold stress (25 °C) and heat stress (37 °C) for 4 hours revealed that *abo1*∆ mutant cells poorly adapt to temperature changes compared to wild-type cells. The double mutants *abo1∆clr3*∆, *abo1∆clr4*∆, and *abo1∆swi6*∆ had a synergistic increase in dead cells under temperature stress compared to the normal growth temperature. The negative genetic interactions of *abo1* and *clr3*/*clr4*/*swi6* suggest that Abo1 is involved in some aspect of heterochromatin assembly.

### Deletion of *abo1* alters H3K9me2 and H3K9me3 levels and causes silencing defects in subtelomeric and pericentromeric heterochromatin regions

**Figure 2:**
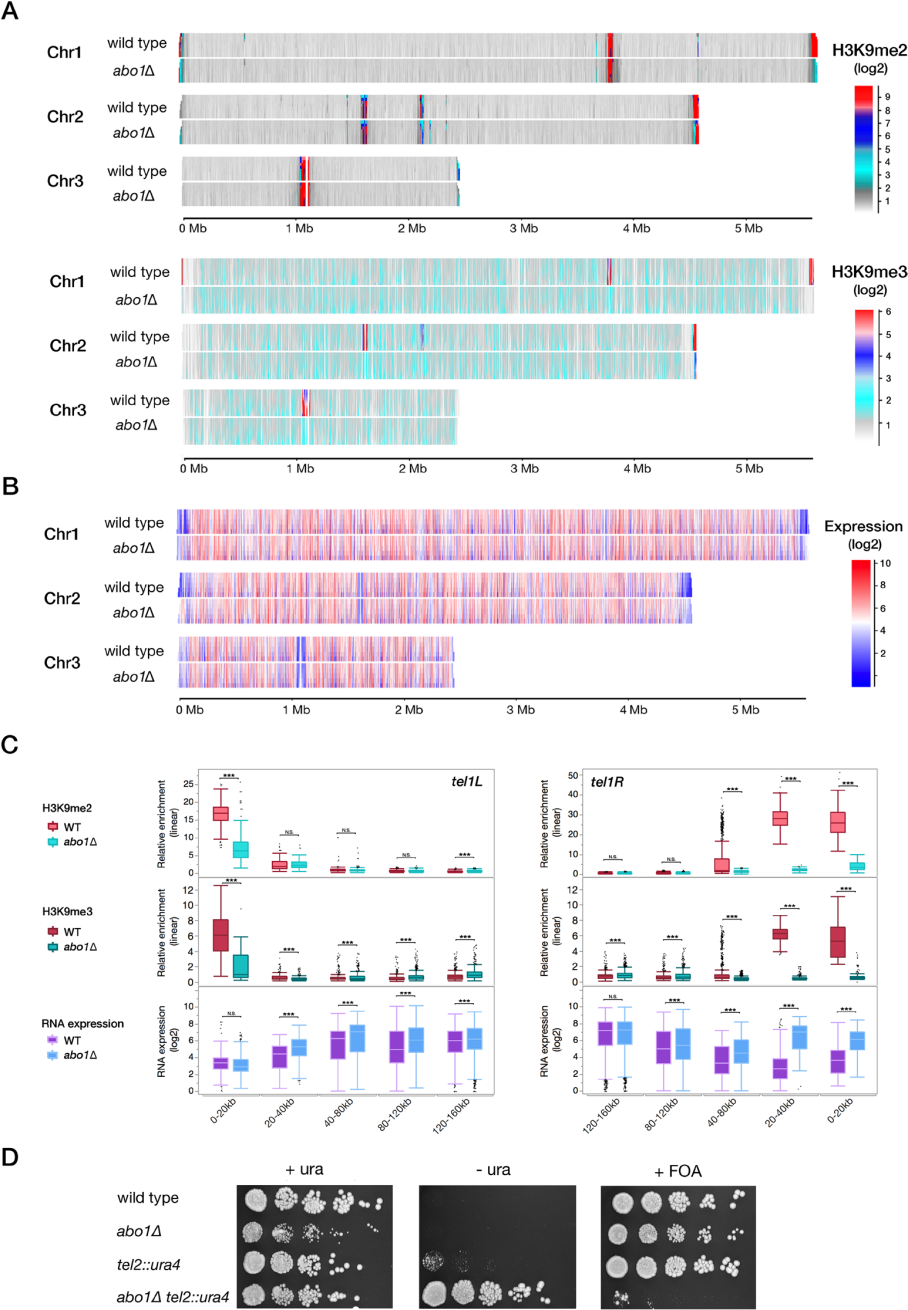
Deletion of *abo1* changes H3K9me2 and H3K9me3 enrichment and causes silencing defects in pericentromeric and subtelomeric heterochromatin regions. (**A**) Domainogram of H3K9me2 enrichment (up-panel) and H3K9me3 (down-panel) on three chromosomes in wild-type and *abo1∆* cells grown at 30 °C. The enrichment is represented by log2 values as indicated by the colours, ranging from weak (grey) to strong (red). (**B**) Domainogram of RNA expression at 30 °C in wild-type and *abo1∆* cells. The Hu2185 and Hu2318 strains were used. The expression is represented by log2 values indicated by the colours, ranging from weak expression (blue) to high expression (red). (**C**) Box plots of the left and right subtelomere regions of chromosome I, showing H3K9me2 enrichment (upper panel), H3K9me3 enrichment (middle panel) and gene expression levels (lower panel) in wild-type cells and *abo1∆* cells grown at 30 °C. The plots represent log2 values. (**D**) Pictures of spotting assays using wild-type and *abo1∆* cells with the *ura4* gene reporter located in the Tel2L region. Cells were grown for 4 days at 30 °C in PMG + uracil, PMG -uracil, and PMG + 5-FOA as indicated. The following strains were used: Hu2185, Hu2318, FY1862, and Hu3030. All ChIP –seq experiments were performed in at least two independent bio-replicates.Error bars indicate standard error of the mean. *p < 0.1, **p < 0.05, ***p < 0.01, two-tailed unpaired t-test. N.S., not significant (i.e., p > 0.1).

Based on previous observations of *abo1∆* silencing defects^38^, and to further investigate the genome-wide role of Abo1 in heterochromatin formation and its effect on transcription, we performed ChIP-seq of H3K9me2/3 marks (Fig. 2A) and RNA profiling (Fig. 2B). The most striking effects occurred in the subtelomeric regions (Fig. 2A-C). For example, at subtelomeres in chromosomes I and II, the H3K9me2 and H3K9me3 levels respectively decreased at least 4-fold and 3-fold in *abo1∆* compared to wild-type cells. De-repression of RNA levels was also observed in the same regions (Supplementary Fig. S1). This effect was especially pronounced in the regions 20 to 40 kb from the chromosome ends (Fig. 2C; Supplementary Fig. S1). These regions are strongly enriched in H3K9me2/3 and Swi6 in wild-type cells^41^. In the left subtelomeric region of chromosome II, the H3K9me2 and H3K9me3 levels were not affected. At chromosome III, the H3K9me2 levels were increased only within 20 kb from the right end, and RNA expression in that area was generally not affected by deletion of Abo1 (Supplementary Fig. S1). Subtelomeric regions of chromosome III are differentially regulated from other subtelomeres, as they contain the rDNA loci.

We further examined the silencing in subtelomeres of *abo1*-deleted cells using the *ura4*^+^ reporter gene inserted in the left subtelomere of chromosome II (*tel2L::ura4*). Silencing of *tel2L::ura4* was examined by the growth of colonies in media without uracil and media containing FOA. In *abo1*-deleted cells, silencing of *tel2L::ura4* was clearly reduced, confirming that Abo1 is required for gene silencing in subtelomeric regions (Fig. 2D).

Our genome-wide mapping of H3K9me2 corroborates the discrete change in *abo1*∆ cells at the pericentromeric regions presented in the supplementary data by Gal *et al*.^38^.as in the *otr1R* region in *abo1∆* cells (Supplementary Fig. S2). A significant reduction of H3K9me3 marks was observed in pericentromeric *otr* regions of all three chromosomes contrasting with H3K9me2 alteration in the same regions, as in *otr1*R region in *abo1∆* cells (Supplementary Fig. S2). In addition, RNA profiling analysis showed that the *otr1R* region was 1.4-fold de-repressed in *abo1*Δ cells compared to wild-type (Supplementary Fig. S3C). As the *abo1*∆ mutant was temperature-sensitive, we examined whether Abo1 is linked to changes in chromatin expression. However, comparisons of genome-wide expression in *abo1-*deleted cells did not reveal notable differences in the silencing of heterochromatic regions at different temperatures (Supplementary Fig. S3A-C). Thus, the silencing defects observed in *abo1∆* cells appear to be mostly independent of growth temperature.

### Abo1 deletion results in a similar phenotype as Clr4 point mutation

**Figure 3:**
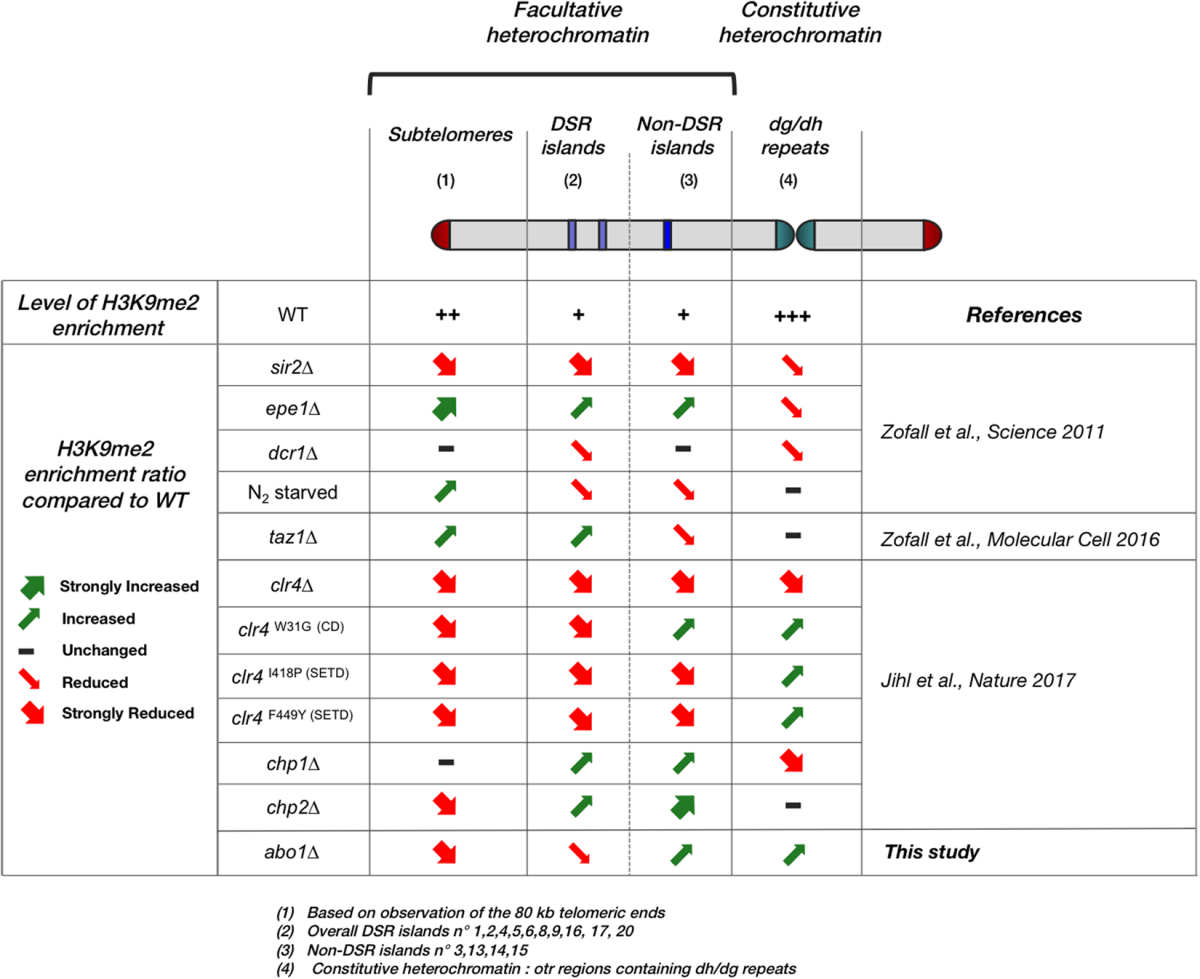
Cells with *abo1* deletion present a similar phenotype as the *clr4* point mutation. The tables summarize changes in H3K9me2 levels in fission yeast cells with the indicated mutant backgrounds compared to wild-type. Small arrows represent a partial or no reduction/increase in H3K9me2 levels in the designated chromatin regions, whereas large arrows indicate a stronger reduction in most of the target compared to the wild-type. Observations were made on the effects based on the mean value of the corresponding region compared to the wild type of extracted published or present data or showed by the authors themselves. H3K9me2 levels of telomeric heterochromatin and islands are shown in Supplementary Data S1, S2, S4, S5, S6, S7, S8. The data were extracted from the publications indicated in the right column.

To search for the heterochromatin assembly mechanism involving Abo1, we extracted published H3K9me2 ChIP-seq data from several different mutant strains^19,22,42^. In addition to the Clr4 mutant strains (*clr4∆*, *clr4*^I418P^, *clr4*^F449Y^, *clr4*^W31G^), other mutations affecting heterochromatin-associated factors, such as the deacetylate Sir2, putative histone H3 demethylase Epe1, siRNA-producing enzyme Dcr1, telomere binding protein Taz1, and HP1 family members Chp1 and Chp2, were included. The effects of these mutations on H3K9me2 levels in the different heterochromatin regions were compared to the H3K9me2 levels in cells lacking Abo1 (Fig. 3; Supplementary Figs. S4-S8). These comparisons revealed a similarity between the *clr4*^W31G^ strain and *abo1∆* cells. For example, in the *otr1R* region, H3K9me2 levels were increased but H3K9me3 levels were reduced compared to wild-type in both *abo1*∆ and *clr4*^W31G^ mutant cells (Supplementary Fig. S2, middle panel; Supplementary Fig. S6, top panel)^43^, which suggested that *abo1* deletion may also disrupt the transition of H3K9me2 to H3K9me3 as *clr4*^W31G^ mutation.

### Abo1 is required for the transition of H3K9me2 and H3K9me3 in pericentric and telomeric heterochromatin

**Figure 4:**
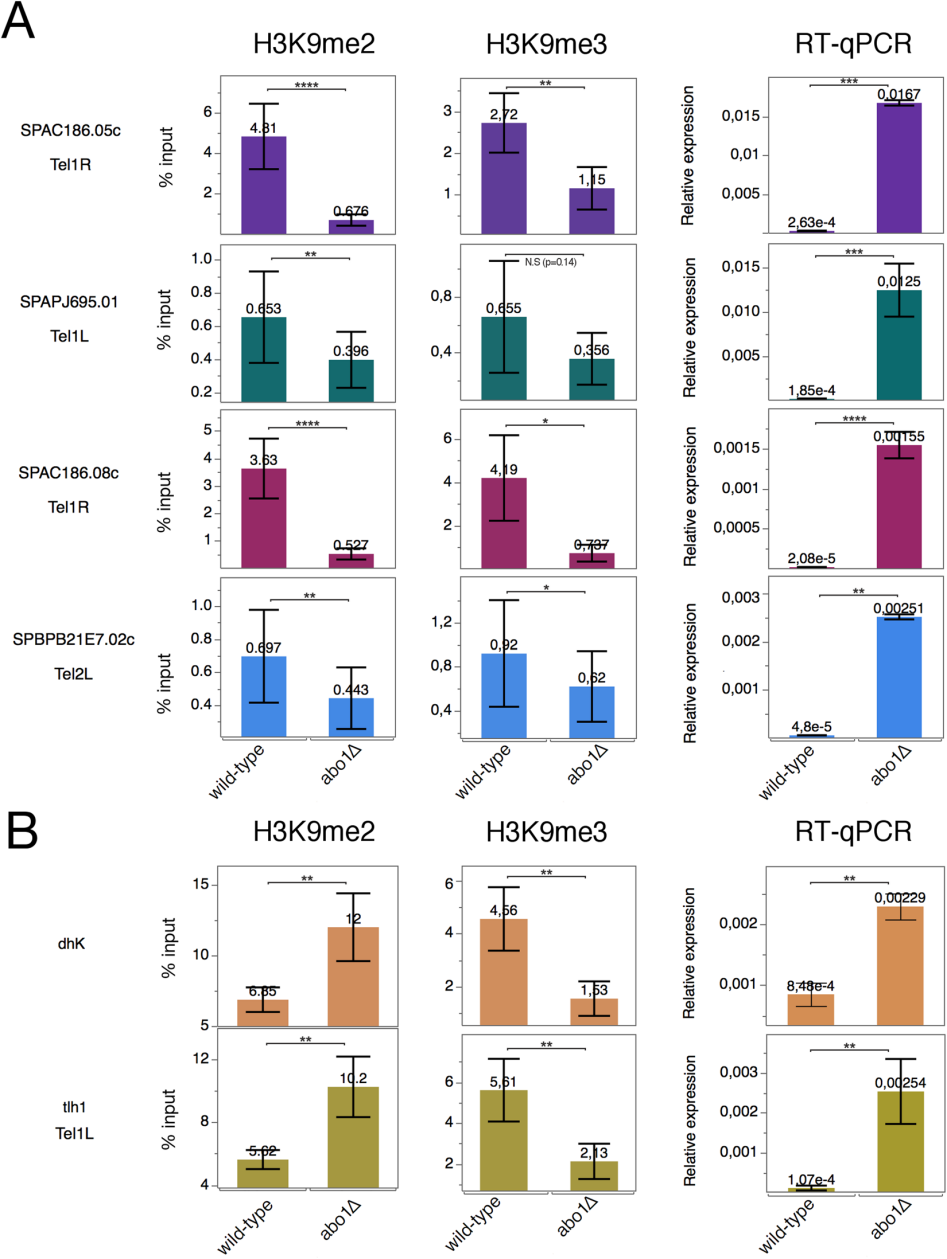
Differential requirement for Abo1 in H3K9me2 and H3K9me3, and silencing of gene expression in subtelomeric and pericentromeric regions. Bar diagrams showing the ChIP and RT-qPCR results. The Hu2185 and Hu2318 strains were used. (**A**) Analysis of four genes located in subtelomeric regions: H3K9me2 (left panels), H3K9me3 ChIP-qPCR (middle panels), and RT-qPCR (right panels). (**B**) Analysis of *dh* repeats located in pericentromeric regions and the Tel1L (*tlh1)* region: H3K9me2 ChIP-qPCR (left panels) and RT-qPCR (right panels). All qPCR experiments were performed in at least three independent experiments. Error bars indicate standard error of the mean. *p < 0.1, **p < 0.05, ***p < 0.001, two-tailed unpaired t-test. N.S., not significant (i.e., p > 0.1).

To confirm our observation on the role of Abo1 in histone methylation, we validated H3K9me2/me3 enrichment by ChIP-qPCR and expression by RT-qPCR of several genes located at subtelomeres, in the pericentromeric *dh* repeats, and at *tlh1*, a gene located near the end of chromosome I that contains *dg-dh*-like repeats (Fig. 4). The four genes located in the subtelomeric regions Tel1R and Tel1L exhibited robust methylation levels in wild-type and a clear reduction of both H3K9me2 and H3K9me3 in *abo1∆* cells (Fig. 4A). All four genes that were tested had 100-fold increased expression in *abo1∆* cells (Fig. 4A, right panel). In contrast, at pericentromeric repeats *(dhK*) and the *tlh1* locus, *abo1*∆ cells had increased H3K9me2 enrichment and reduced H3K9me3 compared to wild-type (Fig. 4B). These changes were accompanied by silencing defects (Fig. 4B, right panel). These observations corroborate the role of Abo1 in mediating the transition from H3K9me2 to H3K9me3, specifically in heterochromatin at telomeres and in centromeric repeat regions. However, subtelomeric genes require Abo1 for both H3K9me2 and H3K9me3 (Fig. 4A), possibly reflecting different Clr4 recruitment mechanisms compared to telomeric (*tlh1*) and centromeric (*dhK*) repeat regions (see Discussion).

### Abo1 is required to maintain H3K9me2 and H3K9me3 levels in DSR islands without any effect on gene expression

**Figure 5:**
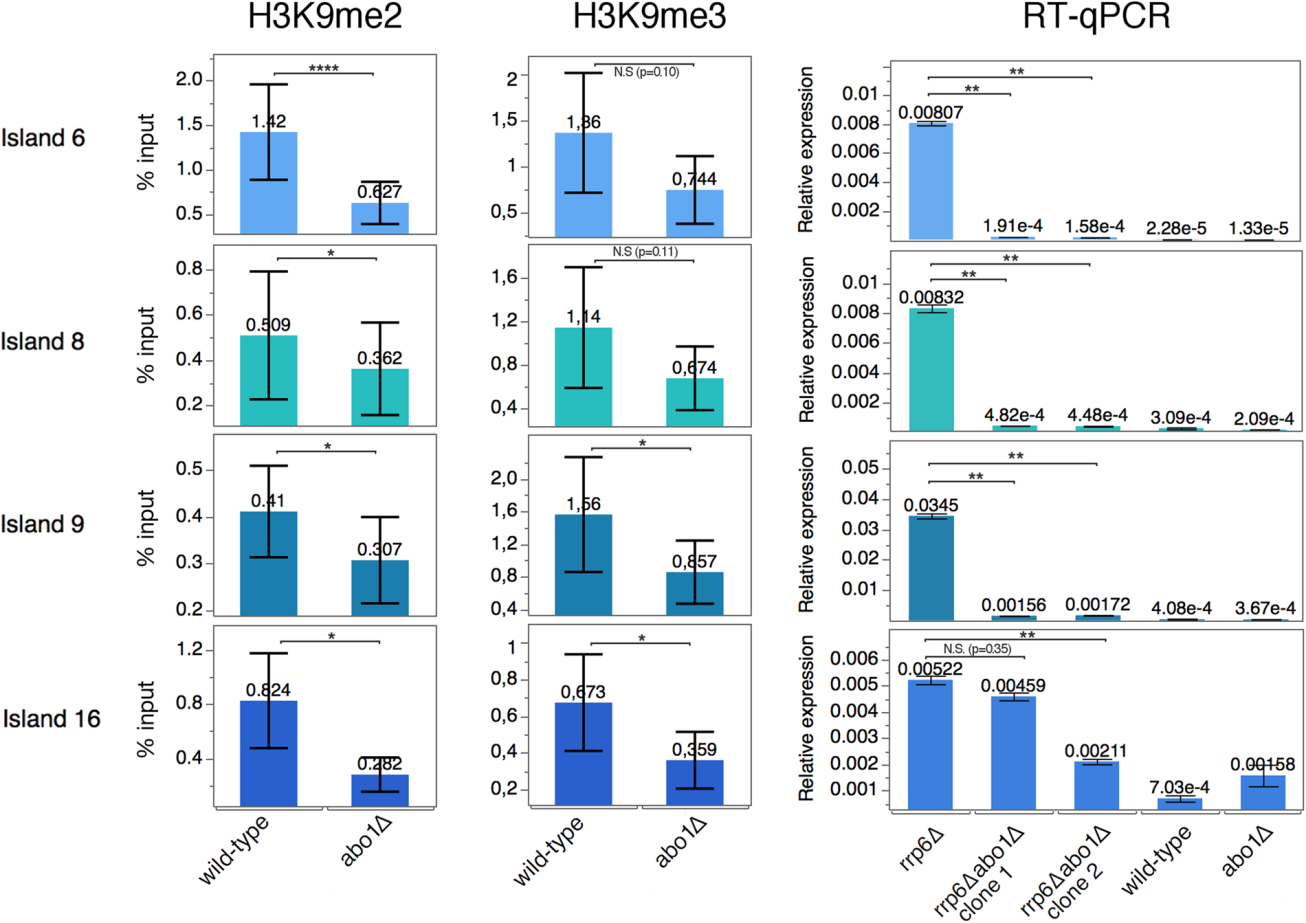
Loss of Abo1 does not affect silencing in DSR islands despite decreased H3K9me2 and H3K9me3 levels. Bar diagrams showing the ChIP and RT-qPCR results for four different heterochromatin DSR islands: Island6 (*ssm4*), Island8 (*mcp5*), Island9 (*mei4*), and Island16 (*pvg4*). H3K9me2 ChIP-qPCR (left panels), H3K9me3 ChIP-qPCR (middle panels), and RT-qPCR (right panels). The genotypes are indicated (wild-type, *abo1∆*, and *rrp6∆*). The following strains were used: Hu2185, Hu2318, Hu2464, and Hu3031 (clone1 and clone2). ChIP-qPCR experiments were performed in at least three independent experiments. RT-qPCR of *abo1∆rrp6∆* was performed in two independent colonies. Error bars indicate standard error of the mean. *p < 0.1, **p < 0.05, ***p < 0.001, two-tailed unpaired t-test. N.S., not significant (i.e., p > 0.1).

Next, we analysed the role of Abo1 in DSR islands by comparing our *abo1*∆ ChIP-seq results to publicly available data^19,42^. The average H3K9me2 levels in *abo1*∆ and *clr4*^W31G^ cells exhibited a decreased level of H3K9me2 in 9 of 10 DSR islands, whereas the levels of H3K9me2 were increased in 3 of 4 non-DSR islands (Fig. 3, Supplementary Fig. S8A). The reduced H3K9me2 levels were confirmed by ChIP-qPCR at several DSR islands (Fig. 5, left panel). H3K9me3 levels were also significantly affected by *abo1* deletion (Fig. 5, middle panel). Next, we analysed the expression of these islands in *rrp6∆abo1∆* cells. The rrp6^+^ gene was deleted to abolish RNA degradation of the DSR genes^19^. Surprisingly, despite the loss of the H3K9me2/me3 heterochromatin markers, deletion of *abo1* led to reduced expression of DSR islands in *rrp6∆abo1∆* compared to *rrp6∆* cells (Fig. 5, right panel). These results show that Abo1 is required for the establishment of heterochromatin and contributes to the transition of H3K9me2 to me3 at DSR islands. Furthermore, Abo1 somehow negatively affects the transcription or stability of DSR island mRNA (see Discussion).

### Clr4 and H3K9 methylation are needed for heterochromatin association of Abo1

Attempts were also made to address whether Abo1 directly interacts with Clr4 by performing Abo1-TAP purification using mass spectrometry. However, no trace of Clr4 was found, suggesting an indirect interaction between Clr4 and Abo1 (Supplementary Fig. S11). We also analyzed at the data from Iglesias *et al*.^44^ who recently determined the Swi6-associated heterochromatin proteome using a method of native chromatin domains coupled with quantitative mass spectrometry (nChIP-MS). In *S. pombe*, Abo1 was found in association with Swi6 that specifically bind domains containing H3K9me^25^ with a similar enrichment level (23 fold, p-value 1.15 10^−2^) (Supplementary Fig. S12B) compared to heterochromatin core proteins. Similar results were found for the closely related species *S. japonicus* that diverged from *S. pombe* approximately 30 million years ago (420 fold, p-value 1.19.10^−2^). Interestingly, when Swi6 nChIP-MS was performed in the clr4 catalytically dead (*clr4*∆CD) and H3K9R substitution mutants, Abo1 was not found (Supplementary Fig. S12B). We also checked in their data if Abo1 Swi6 heterochromatin association could depend on others heterochromatin components required for silencing such as Chp2, Dcr1, Ago1, Sir2, Epe1. None of these factors affects the association between Abo1 with FLAG-Swi6 (Supplementary Fig. S12C). Thus, we conclude from this analysis that Abo1 is associated with Swi6 chromatin and this is dependent of H3K9me and Clr4.

### Abo1 is required to stabilise Clr4 recruitment at heterochromatin

**Figure 6:**
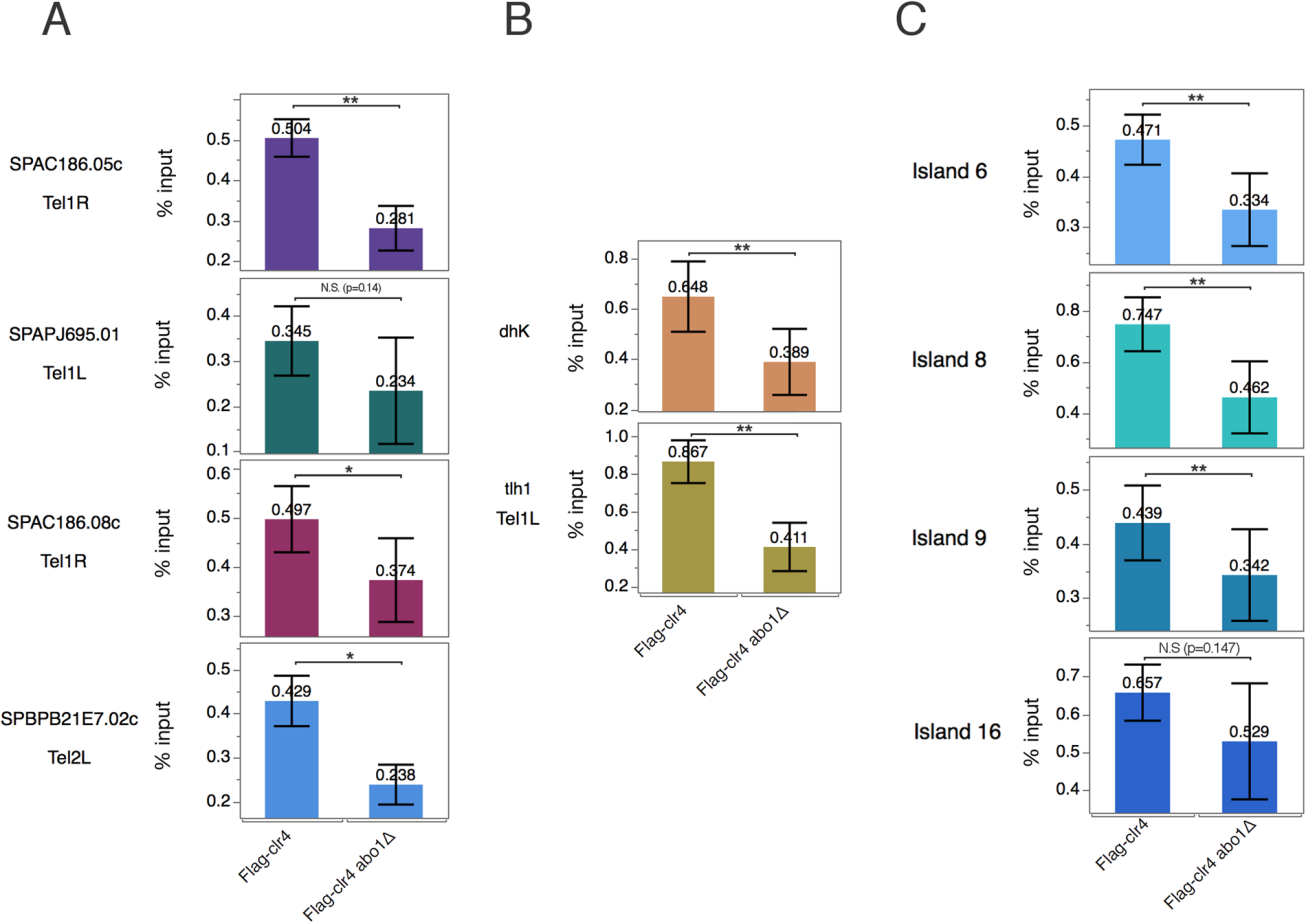
Deletion of Abo1 leads to reduced enrichment of Clr4 in all heterochromatin regions. Bar diagrams showing the Flag ChIP-qPCR results. The SPJ390 and Hu3040 strains were used. **(A)** Analysis of four genes located in subtelomeric regions. The locations of genes are indicated. (**B**) Analysis of *dh* repeats located in pericentromeric regions and the Tel1L (*tlh1)* region. (**C**) Analysis of four different heterochromatin DSR islands: Island6 (*ssm4*), Island8 (*mcp5*), Island9 (*mei4*), and Island16 (*pvg4*). All qPCR experiments were performed in at least three independent experiments. Error bars indicate standard error of the mean error. *p < 0.1, **p < 0.05, ***p < 0.001, two-tailed unpaired t-test. N.S., not significant (i.e., p > 0.1).

Using ChIP-qPCR, we compared Clr4 enrichment at heterochromatin regions between the Clr4 flag-tagged and Clr4 flag-tagged *abo1*∆ strains. At the four genes tested in the subtelomeric regions, we observed a marked decrease in Clr4 occupancy in *abo1* deletion (Fig. 6A) consistent with the decrease of H3K9me3 observed in the same loci in *abo1∆* cells (Fig. 5A). At the centromeric (*dhK*) and telomeric repeats (*tlh1*), Clr4 occupancy was also significantly reduced in *abo1*∆ cells (Fig. 6B). A similar decrease in Clr4 was observed at DSR islands (Fig. 6C). Generally, these results support the role of Abo1 to stabilise Clr4 recruitment to allow the H3K9me2-H3K9me3 transition at different heterochromatin regions.

## Discussion

**Figure 7:**
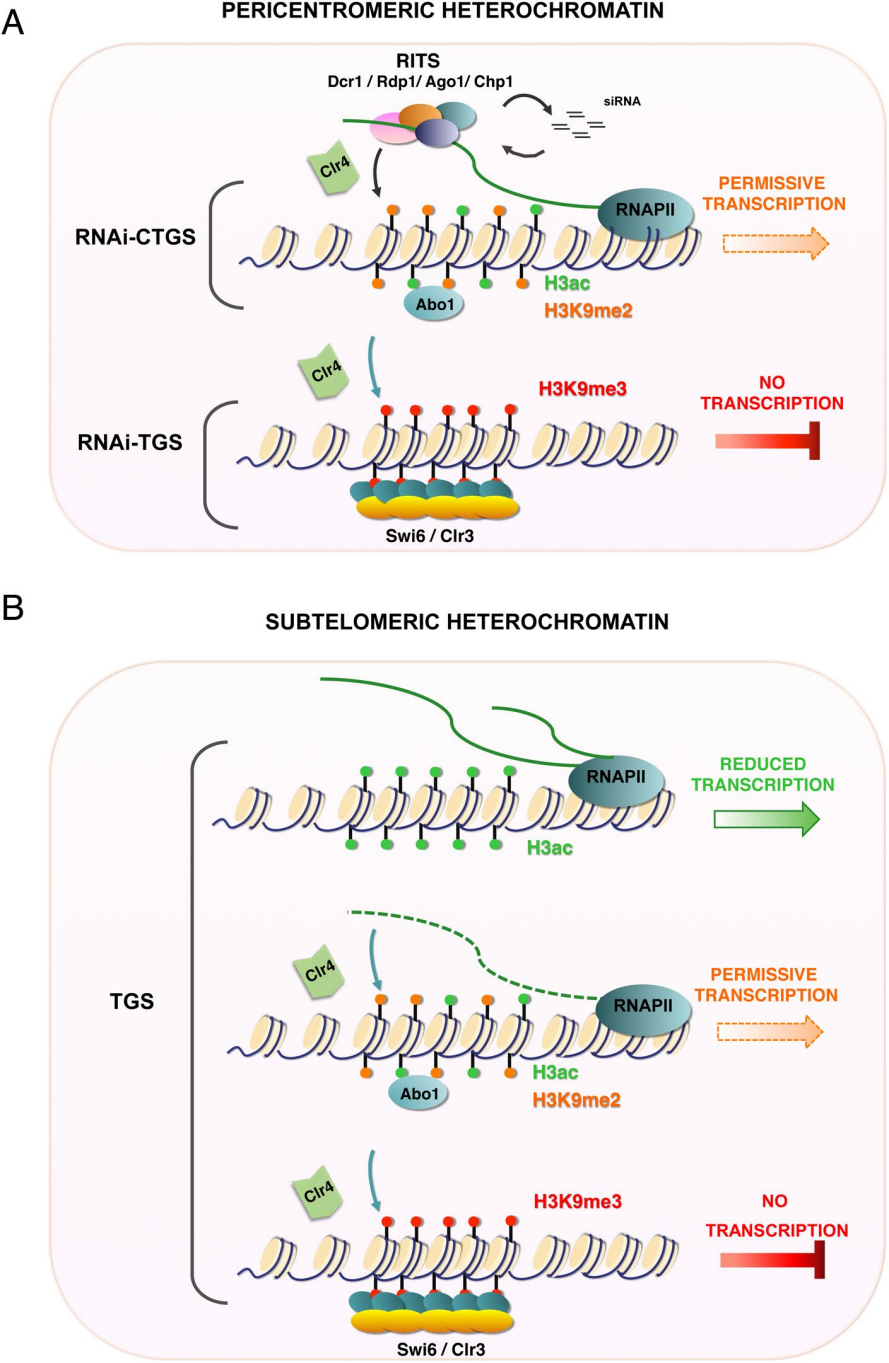
Model of the proposed role of Abo1 in heterochromatin in *S. pombe*. Schematic models of Abo1 function in constitutive heterochromatin (**A**) and facultative heterochromatin regions (**B**).

The present study showed that Abo1 is required for the proper regulation of chromatin silencing and maintenance of the H3K9me2/3 levels at both facultative and constitutive heterochromatin regions. Until recently, it has generally been thought that the H3K9me2 marker in *S. pombe* heterochromatin inversely correlates with expression^45^. In 2017, a study in *S. pombe* revealed that H3K9me2 and H3K9me3 have distinct roles in gene silencing, with H3K9me2 being permissive for transcription^22^. Our work identifies Abo1 as a new factor involved in the H3K9 methylation process. In pericentromeric heterochromatin, silencing occurs in two steps: RNAi co-transcriptional transcriptional gene silencing (RNAi-CTGS) followed by transcriptional gene silencing (RNAi-TGS)^22^. In the RNAi-CTGS step, the *dg*-*dh* repeats can still be transcribed, and siRNAs are generated by Dicer and Ago1, consequently activating the RITS complex (Fig. 7A)^46–48^. Next, the siRNA-activated RITS complex helps recruit the methyltransferase Clr4 to perform di-methylation of H3K9^49,50^. At this stage, H3K9me2 and euchromatic H3 acetylation (H3ac) are present, allowing for a low level of transcription. In the RNAi-TGS step, H3K9 is tri-methylated, Swi6 binds, and the Clr3 deacetylase enzyme removes acetylation of H3 to stop transcription (Fig. 7A). We hypothesise that Abo1 appears in the first step when H3K9me2 has been added by Clr4. Our finding that Abo1 association with heterochromatin depends on Clr4 and H3K9 methylation is consistent with this idea (Supplementary Fig. S12). Furthermore the reduction of Clr4 recruitment that we observe at *dhK* and *tlh1* in *abo1∆* strains suggests that Clr4 is stabilised by Abo1 (Fig. 6B).

In contrast, at genes in subtelomeric regions (Fig. 7B), di-methylation on H3K9 does not require the siRNA-RITS complex. Here, Abo1 may help to recruit Clr4 promoting both H3K9me2 and H3K9me3, leading to TGS. The data in Fig. 6A, showing that Clr4 binding to subtelomeres depends on Abo1, supports this notion. At both pericentric and telomeric regions, the physical interaction observed between Abo1 and Swi6^51^ may contribute to the assembly of heterochromatin.

Interestingly, the reduced expression and H3K9me2 levels at the DSR islands (Fig. 5) suggest that Abo1 plays a different role with Clr4 in this facultative heterochromatin. A recent study showed that, despite its presence at DSR genes, Clr4 is not involved in regulating mRNA levels at normal growth temperature, whereas Set1-mediated deacetylation by Clr3 and other HDACs plays a key role^52^. However, loss of Clr4 in cells cultivated at 18 °C alters meiotic gene silencing but also the assembly of new facultative heterochromatin islands indicating a critical role for Clr4 at lower temperature^53^. Since Abo1 deletion have been show to affect the global gene expression and nucleosome occupancy *in vivo*^38^, we cannot rule out the possibility that Abo1 acts across the genome affecting indirectly the gene expression. Whether Abo1 modulates Set1 and HDAC activity at facultative heterochromatin islands or mediates their silencing through the Clr4 recruitment at low temperature remains to be determined.

One possibility is that the observed effect on methylation patterns in the tested strains could be linked to nucleosome dynamics. We first looked at nucleosome positioning using data from Gal *et al*.^38^ in the same regions analysed in our study. However, no obvious changes were observed at either the positioning or nucleosome levels in *abo1*∆ compared to wild-type cells at the subtelomeric, pericentromeric, and non-DSR island regions (Supplementary Fig. S9). Absence of change was also observed when the H3 occupancy was tested for the same regions using ChIP-qPCR *abo1*∆, *clr4*W31G, and *abo1*∆ *clr4*W31G strains (Supplementary Fig. S10). Together, both of these results suggest that nucleosome dynamics defects are not the main cause of H3K9me transition defects in *abo1*∆ cells.

In *C. elegans*, the heterochromatin includes telomeric and subtelomeric regions, as well as centromeres and repetitive DNA transposons ^54,55^. Similar to fission yeast, these heterochromatin regions are enriched in H3K9 methylation^54^, and an HP1-like protein, HPL-2, binds to regions correlating with H3K9me2/3 enrichment^55,56^. The analysis of H3K9me2 and H3K9me3 patterns has revealed that these markers can be enriched in different chromatin regions associated with distinct functions in *C. elegans*^20^. H3K9 methylation occurs step-wise by different enzymes: MET2 for mono-/di-methylation and SET25 for tri-methylation. The latter step brings heterochromatin to the nuclear periphery and leads to TGS^21^. In *S. pombe*, whether tri-methylation is coupled to peripheral localisation in the nucleus remains to be tested. Given our findings for Abo1, it would be interesting to test whether an ATAD2 homologue in *C. elegans* modulates the activity of MET2 or SET25. A candidate for this function could be the ATAD2 homologue LEX-1, as it has been reported to modulate gene expression at repetitive DNA sequences^57^.

The exact function of the human Abo1 homologue ATAD2 in both cancer and non-cancer cells remains unclear. However, ATAD2 has been proposed to be involved in the regulation of many key factors, such as E2F, Myc, and EZH2, as well as downstream crosstalk with P53/P21 pathways^58–61^. In embryonic stem cells, knockdown of ATAD2 leads to an overall increase in nucleosome density and reduced histone turnover^62^. In breast cancer, ATAD2 has higher affinity for heterochromatin than euchromatin during S phase^63^. Although it is conceivable that the mechanistic role of AAA family proteins is conserved in eukaryotes, future studies need to address the putative role of human Abo1 homologues in heterochromatin assembly.

## Materials and Methods

### Schizosaccharomyces pombe strains and growth conditions

Culture, genetic manipulation, and fission yeast strains are described in Supplementary Table S2. Standard YES medium was used for cell culture. Standard fission yeast genetic techniques and media were used according to Moreno *et al*.^64^. Yeast cells were grown to mid-logarithmic phase. Cells were then counted and diluted to the concentration of 1.25 × 10^6^ cells/ml. Five-fold serial dilution was performed. A total of 5 μl of each dilution was spotted on complete PMG media plates containing 1 g/L FOA (5-fluorouracil-6-carboxylic acid monohydrate) and PMG media lacking uracil. The plates were incubated at 30 °C.

### FACS analysis

*S*. pombe cells were grown to log phase in triplicate for fluorescence-activated cell sorting (FACS). Cells were then washed with 1x PBS and subsequently resuspended in 50 mM sodium citrate (pH 7) with 50 μg/ml propidium iodide (PI) for 30 min in the dark at room temperature (Sigma Aldrich). Cells were analysed immediately using a flow cytometer (Cytoflex, Beckman Coulter). Cells were sorted by forward (FSC) and side (SSC) light scattering. After gating for 20,000 single cells using FSC and SSC, dead cells stained with PI were counted through the FL2- channel.

### RNA extraction and RNA microarray

Wild-type (Hu2185) and *abo1∆* strains (Hu2318) were cultured to mid-log exponential phase and continuously induced at 25 °C, 30 °C, or 37 °C for 4 hours. The cells were then harvested by centrifugation at 3000 rpm and lysed by incubation with TES buffer (10 mM Tris-HCl, pH 7.5, 10 mM EDTA, and 0.5% SDS). RNA was extracted using the hot acid-phenol method. Biological duplicates of RNA samples were sent for hybridisation to Gene Chip *S. pombe* Tilling 1.0FR Arrays (Affymetrix, Santa Clara, CA, USA) at the Affymetrix core facility (BEA) of Karolinska Institutet. For RNA microarray analysis, the signal raw data files were normalised using Affymetrix Tiling Analysis Software (TAS). The normalised microarray data were mapped to the *S. pombe* genome (Sanger 2007). Microarray results were visualised using SeqMonk software (https://www.bioinformatics.babraham.ac.uk/projects/seqmonk/). The microarray data can be accessed at GEO accession GSE 125910.

### RT- qPCR

For each cell culture, 1.0 μg of the extracted RNA was treated with DNase I (18068015, Thermo Fisher) and isolated for reverse transcription by SuperScript™ III First-Strand Synthesis Master Mix (11752050, Invitrogen). RT-qPCR was then applied by using the SYBR™ Green PCR Master Mix (436870, Thermo Fisher). The reactions were performed using an Applied Biosystems® 7500 Real-Time PCR System. The primer sequences are listed in Supplementary Table S3.

### Chromatin immunoprecipitation

Chromatin samples from wild-type (Hu2185), *abo1*∆ cells (Hu2318), Flag-clr4 cells (SPJ390), and *abo1*∆ Flag-clr4 cells (Hu3040) were isolated in duplicate and subjected to ChIP according to Durand-Dubief *et al*.^65^. A total of 50 μg (for ChIP-seq) or 4 μg (for ChIP-qPCR) of H3K9me2 antibody (ab1220, Abcam), 4 μg (for ChIP-seq) or 1 μg (for ChIP-qPCR) of H3K9me3 biotinylated antibody (C1550003, Diagenode), 4 μg of Flag M2 antibody (F1804, Sigma), and 4 μg of H3 antibody (ab1791, Abcam) were added to 30 μl of sheared chromatin (for ChIP-qPCR) or 100 μl of sheared chromatin (for H3K9me2 ChIP-seq) or 200 μl of sheared chromatin (for H3K9me3 ChIP-seq) for each experiment. Protein A Dynabeads (10001D, Invitrogen) were used for H3K9me2 ChIP, and m280 streptavidin Dynabeads (11205D, Invitrogen) for H3K9me3 ChIP with an additional antibody pre-incubation step. ProteinA/G Magnetic beads (88802, Thermo Scientific) were used for Flag ChIP and H3 ChIP. ChIP DNA was sequenced and the sequencing data processed by the core facility (BEA) of Karolinska Institutet. Processed data were quantified using a read count per million reads normalisation, and then relative enrichment compared to the DNA input using SeqMonk software (https://www.bioinformatics.babraham.ac.uk/projects/seqmonk/). ChIP-DNA was also quantified for quantitative PCR using Applied Biosystems® 7500 Real-Time PCR Systems. SYBR™ Green PCR Master Mix (436870, Thermo Fisher). Primers are listed in Expanded View Table EV2. ChIP-seq data can be accessed at GEO accession GSE125911.

### Synthetic genetics array analysis

A total of 760 haploid *S. pombe* strains harbouring deletions of genes involved in chromatin processes were selected from the Bioneer deletion library V.5^66^ using Gene Ontology terms “chromatin binding”, “DNA binding”,”chromosome binding”, “chromosome”, and “transcription”. These strains have *gene*∆::*Kan*^R^ deletions and carry the auxotrophic markers ade6-M216 (or *ade6-M210*) *ura4-D18* and *leu1-32*. The 760 strains were crossed with the smt0 *abo1*∆::*hyg*^R^ strain (Hu3021), spores transferred in YES media, and successively selected three times on YES with G418 (200 μg/ml) and YES with hygromycin (200 μg/ml). The cross resulted in 716 double mutants carrying a deletion for a gene involved in the chromatin process combined with the *abo1* gene deletion using the RoToR robot (Singer Instruments). For analysis, single and double mutants were grown for 2 days using semi-solid YES media at 30 °C with the 384-plate format in triplicate. Plates were scanned and the colony sizes measured for four replicates and normalised using SGAtools^67^. Next, the colony fitness score was obtained using the normalised colony sizes of the single and double mutants. Negative scores indicate that the double mutant induces an increased fitness defect than expected; conversely, positive scores correspond to greater fitness compared to the control. The scoring distribution of these strains was analysed and a cut-off score of <−0.45 applied.

### Protein purification and mass spectrometry

Six litres of Abo1-TAP (Hu2368) and wild-type cells were cultured to log-phase. Pelleted cells were then ground using the grinding machine (6875 Freezer/Mill® High Capacity Cryogenic Grinder) with liquid nitrogen. The frozen yeast powders were resuspended in lysis buffer (150 mM NaCl, 1% NP-40, 50 mM Tris-HCl). Cell lysates were ultra-centrifuged and the supernatant removed. The supernatant was flowed through pre-washed IgG Sepharose 6 Fast Flow beads (17-0969-01, GE Healthcare). Using TEV protease (AcTEV Protease, 12575-015, NOVEX), TAP-tagged Abo1 protein was eluted (TAP affinity purification protocol according to (http://cshprotocols.cshlp.org/content/2006/1/pdb.prot4153.full)^68,69^. Eluted samples were quantified and sent for mass spectrometry at the chemical core facility in Karolinska Institutet^70^.

## Supplementary information

Supplementary Dataset.

Supplementary information file.
