# Supplementary material for: Abo1 is required for the H3K9me2 to H3K9me3 transition in heterochromatin
Source: Sci Rep. 2020 Apr 8;10:6055. doi: 10.1038/s41598-020-63209-y (PMC7142091; doi:10.1038/s41598-020-63209-y)

Supplementary dataset

Abo1 is required for the H3K9me2 to H3K9me3 transition in heterochromatin

Wenbo Dong[1], Eriko Oya[1], Yasaman Zahedi[1], Punit Prasad[1,2], J Peter Svensson[1], Andreas Lennartsson[1], Karl Ekwall[1], Mickaël Durand-Dubief[1,]*.

[1]Dept of Biosciences and Nutrition, Karolinska Institutet NEO, 141 83 Huddinge, Sweden

[2]Institute of Life Sciences, NALCO Square, *Bhubaneswar,* 751023 Odhisa, India

*Corresponding author :

mickael.durand-dubief@ki.se

Supplementary Table S1

Dong et al.

Gene name	Description	Score	Std
nto1	histone acetyltransferase complex subunit Nto1 (predicted)	-1,08	0,35
pof3	F-box protein Pof3	-0,90	0,03
swd2	Set1C complex subunit Swd2.1	-0,80	0,27
clr3	histone deacetylase (class II) Clr3	-0,75	0,26
rxt3	transcriptional regulatory protein Rxt3	-0,73	0,03
set1	histone lysine methyltransferase Set1	-0,71	0,12
whi5	cell cycle transcriptional repressor Whi5 (predicted)	-0,61	0,15
SPAC57A7.09	ubiquitin-protein ligase E3, human RNF13 family homolog	-0,60	0,07
oga1	Stm1 homolog Oga1	-0,59	0,10
msc1	multi-copy suppressor of Chk1	-0,56	0,21
SPBC32F12.07c	membrane associated ubiquitin-protein ligase E3, MARCH family (predicted)	-0,55	0,11
rad3	ATR checkpoint kinase Rad3	-0,54	0,05
vps8	WD repeat protein Vps8 (predicted)	-0,54	0,10
vgl1	vigilin (predicted)	-0,53	0,05
swi6	chromodomain protein Swi6	-0,52	0,09
fil1	transcription factor, zf-GATA type (predicted)	-0,51	0,11
fml1	ATP-dependent 3' to 5' DNA helicase, FANCM ortholog Fml1	-0,51	0,09
slx4	structure-specific endonuclease subunit	-0,50	0,14
clr2	chromatin silencing protein Clr2	-0,50	0,10
chp2	chromodomain protein 2	-0,49	0,02
sty1	MAP kinase Sty1	-0,49	0,09
clr4	histone H3 methyltransferase Clr4	-0,49	0,05
ubp8	SAGA complex ubiquitin C-terminal hydrolase Ubp8	-0,49	0,06
reb1	RNA polymerase I transcription termination factor Reb1	-0,49	0,10
sea3	ubiquitin-protein ligase E3, coatamer related complex subunit Sea3 (predicted)	-0,48	0,04
prz1	calcineurin responsive transcription factor Prz1	-0,47	0,21
cda1	chitin deacetylase Cda1	-0,47	0,02
atf31	transcription factor Atf31	-0,47	0,04
xrc4	XRCC4 nonhomologous end joining factor Xrc4	-0,46	0,00
chp1	chromodomain protein Chp1	-0,45	0,19

Dong et al.

Strains	Genotypes	Source
Hu2185	*h+ ade6-M210 ura4-D18 leu1-32*	R.Allshire
Hu2318	*h+ abo1::KanMX ade6-M210 ura4-D18 leu1-32*	Bioneer
Hu2545	*h- abo1::hphMX ade6-M210 ura4-D18 leu1-32*	this study
Hu1098	*h- sir2::KanMX ade6-M210 ura4-D18 leu1-32*	Bioneer
FY1862	*h+ ade6-M210 his3-D1 ura4-D18 leu1-32 otr1RSphI::ade6+ his3::Tel(1L) ura4+::Tel(2L)*	R.Allshire
Hu2464	*h- rrp6::KanMX ade6-M210 ura4-D18 leu1-32*	Bioneer
Hu3021	*h- smt0 abo1::hphMX ade6-M210 ura4-D18 leu1-32*	this study
Hu3022	*h+ clr3::KanMX ade6-M210 ura4-D18 leu1-32*	Bioneer
Hu3023	*h+ clr4::KanMX ade6-M210 ura4-D18 leu1-32*	Bioneer
Hu3024	*h+ swi6::KanMX ade6-M210 ura4-D18 leu1-32*	Bioneer
Hu3026	*h? clr3::KanMX abo1::hphMX ade6-M210 ura4-D18 leu1-32*	this study
Hu3027	*h? clr4::KanMX abo1::hphMX ade6-M210 ura4-D18 leu1-32*	this study
Hu3028	*h? swi6::KanMX abo1::hphMX ade6-M210 ura4-D18 leu1-32*	this study
Hu3030	*h? abo1::hphMX ade6-M210 his3-D1 ura4-D18 leu1-32 otr1RSphI::ade6+ his3::Tel(1L) ura4+::Tel(2L)*	this study
Hu3031	*h? rrp6::KanMX abo1::hphMX ade6-M210 ura4-D18 leu1-32*	this study
SPJ390	*mat1Msmt0m leu1-32 his2-ura4D/E ade6-M210 otr::ura4+; Flag-clr4*	S.Jia
Hu3040	*mat1Msmt0m leu1-32 his2-ura4D/E ade6-M210 otr::ura4+; abo1::KanMX; Flag-clr4*	this study

Supplementary Table S3

Names	Sequences
SPAC186.05c_Forward	5'-CCAATATGGACGGGCATCCG-3'
SPAC186.05c_Reverse	5'-AGGGAACAGAAAAGGGGCAG-3'
SPAC186.08c_Forward	5'-ACAGGATTCCCAGCTGAACG-3'
SPAC186.08c_Reverse	5'-ACACTTTGAGGATCAAGGCCA-3'
SPAPJ695.01c_Forward	5'-GCTAACCGGTCAGTATACCACT-3'
SPAPJ695.01c_Reverse	5'-TGGGATCCAAGTCGCTGTTT-3'
SPBPB21E7.02c_Forward	5'-CGTACGTTGCAAACGATGGA-3'
SPBPB21E7.02c_Reverse	5'-GTCGCAAGGCCAATTTCCAG-3'
dhk_Forward	5'-ATAGCGCCACACTTTTGAGC-3'
dhk_Reverse	5'-TGGGCATCAAAGGAATGTTT-3'
Islands6_ssm4_Forward	5'-GACCGCAAGGATGAAACTGG-3'
Islands6_ssm4_Reverse	5'-TCCTTGCAGGCAAAGGTCAA-3'
Islands8_mcp5_Forward	5'-TAAGGACCGATGCGCTTTCA-3'
Islands8_mcp5_Reverse	5'-GACGAGGAATCGAGCAGTCA-3'
Islands9_mei4_Forward	5'-ATGTTGCTGCCGAAGTCTCA-3'
Islands9_mei4_Reverse	5'-TTAGAAGGGGGAAGAGGGGG-3'
Islands16_mbx2_Forward	5'-GGCTTGCCCTCCTTTTCTCT-3'
Islands16_mbx2__Reverse	5'-GTAGGCGGAATAGGTGAGCC-3'
Tlh1_Forward	5'-GACCACAACTTGCGAGAATG-3'
Tlh1_Reverse	5'-GCAATAGTCCTTCGGTATTG-3'

Supplementary Figure S1

Dong et al.

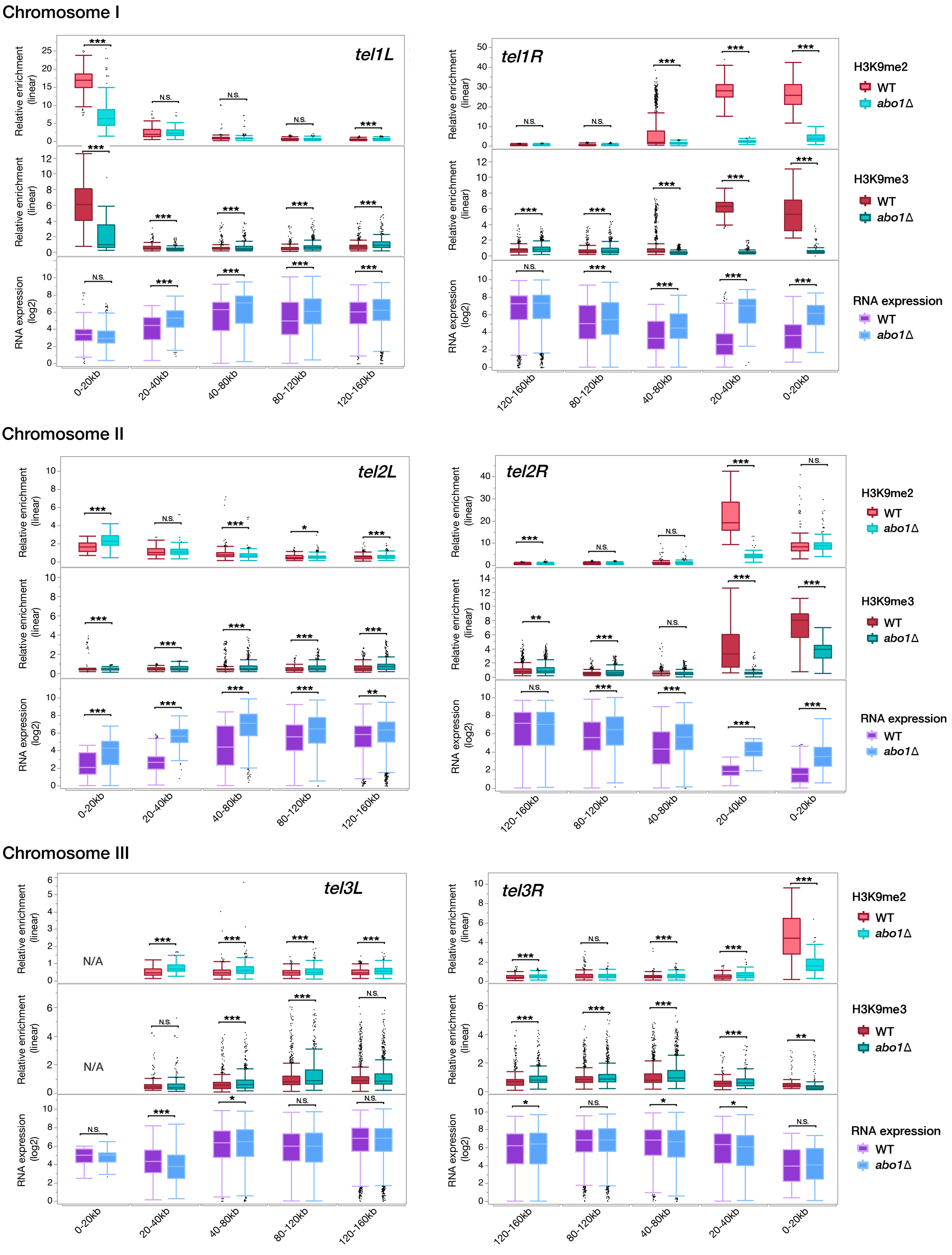

Supplementary Figure S2

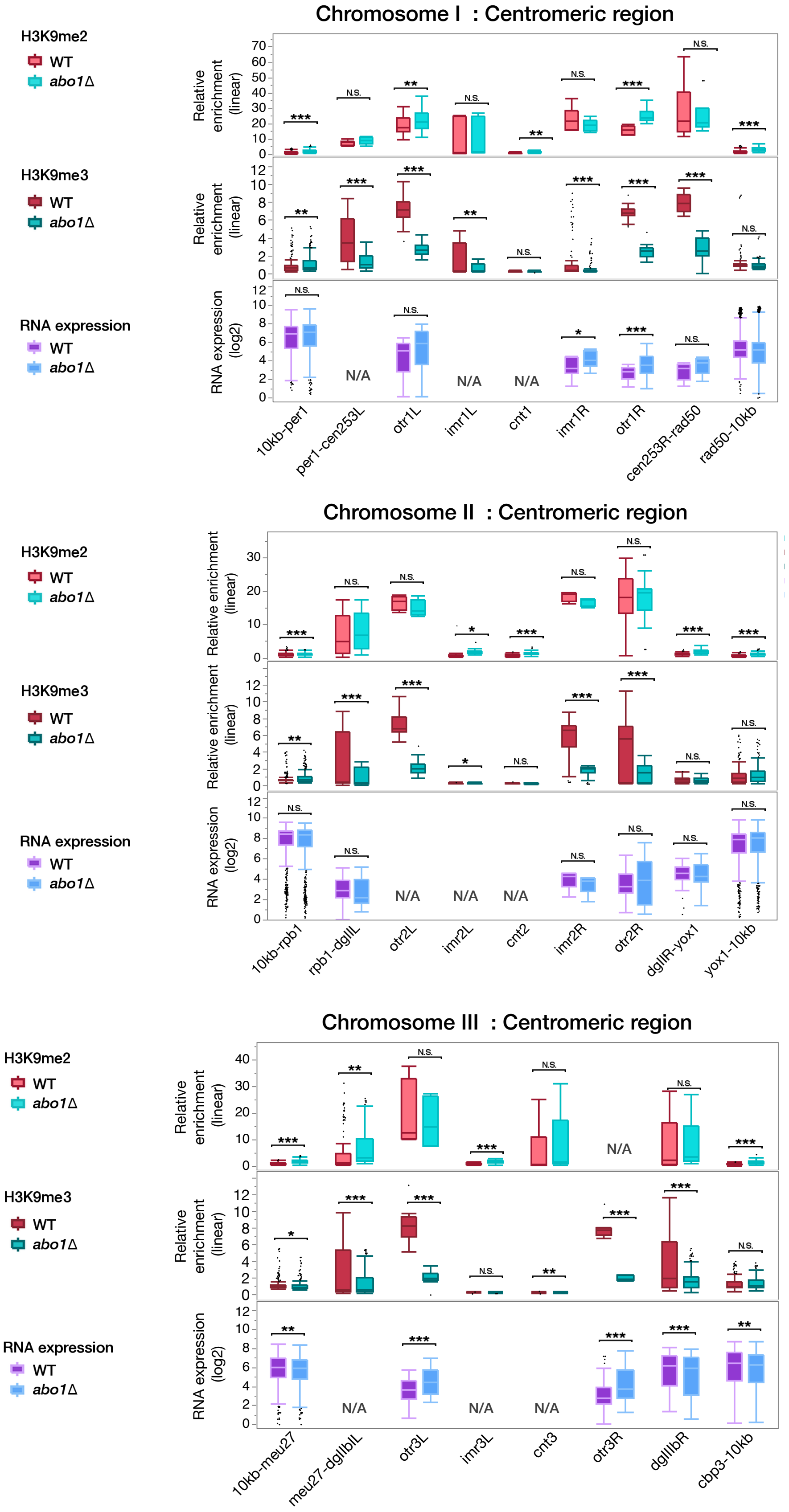

Supplementary Figure S3

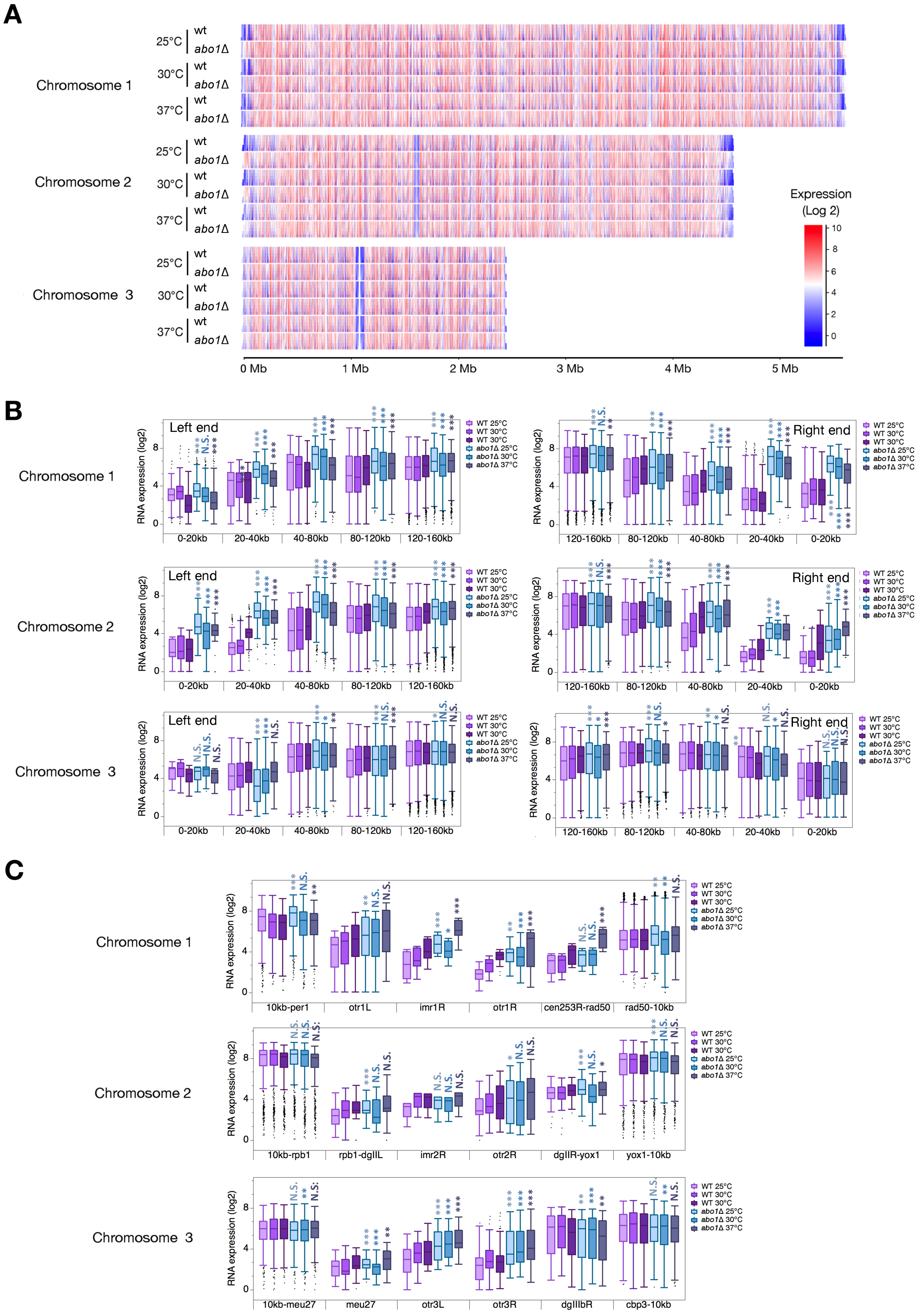

Supplementary Figure S4

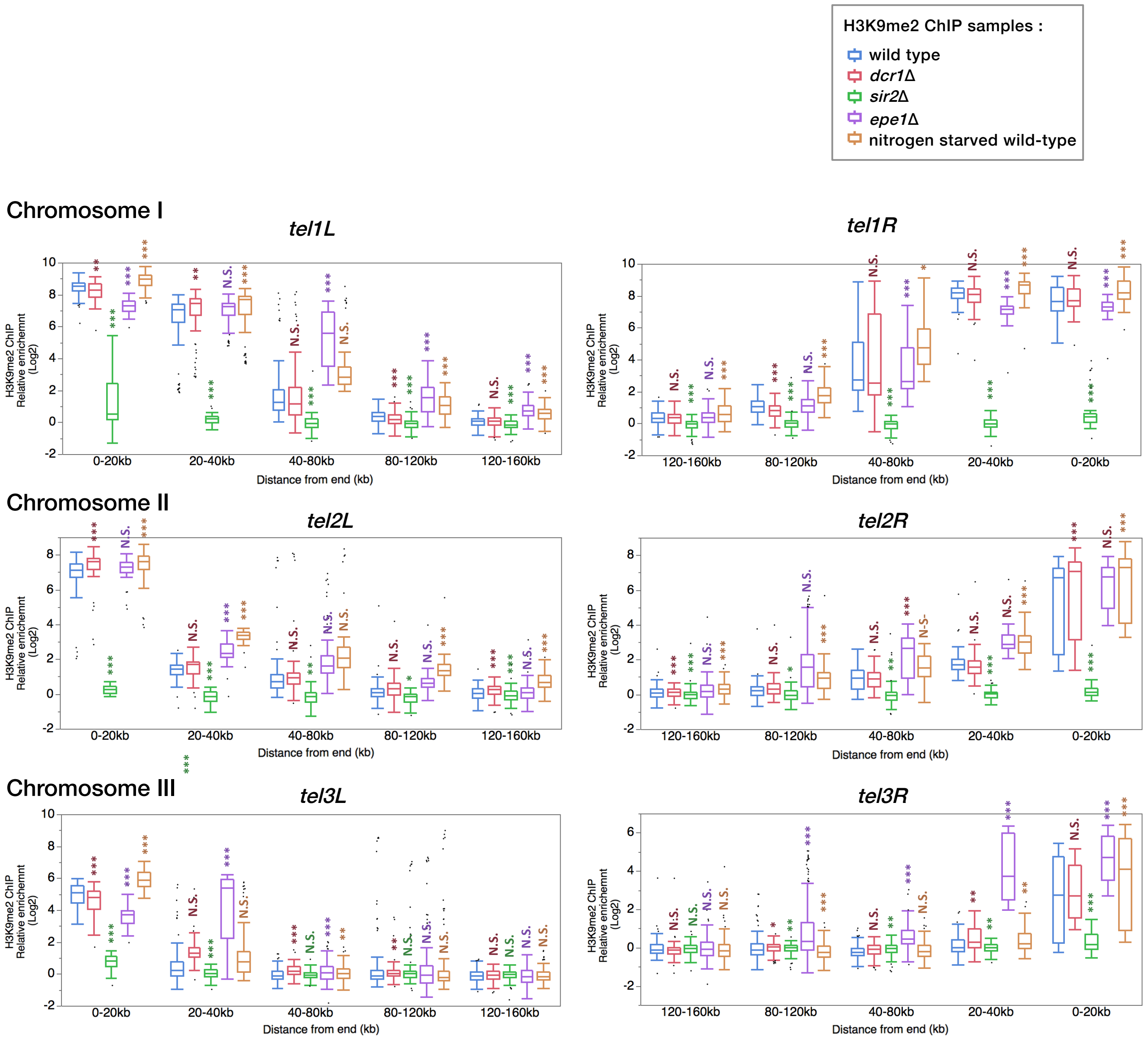

Supplementary Figure S5

Dong et al.

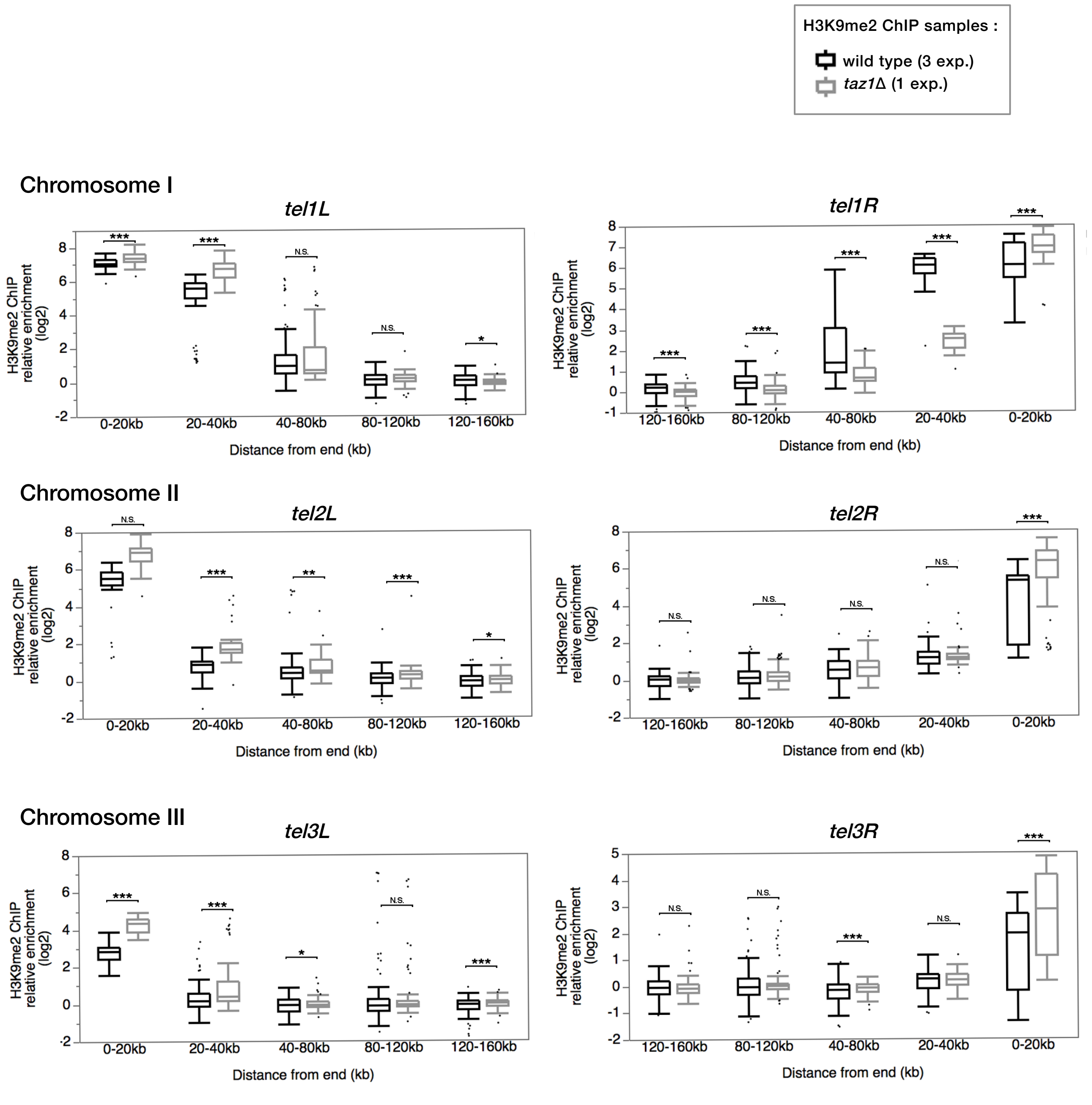

Supplementary Figure S6

Dong et al.

Clr4 point mutations

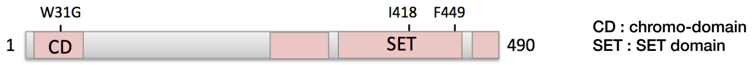

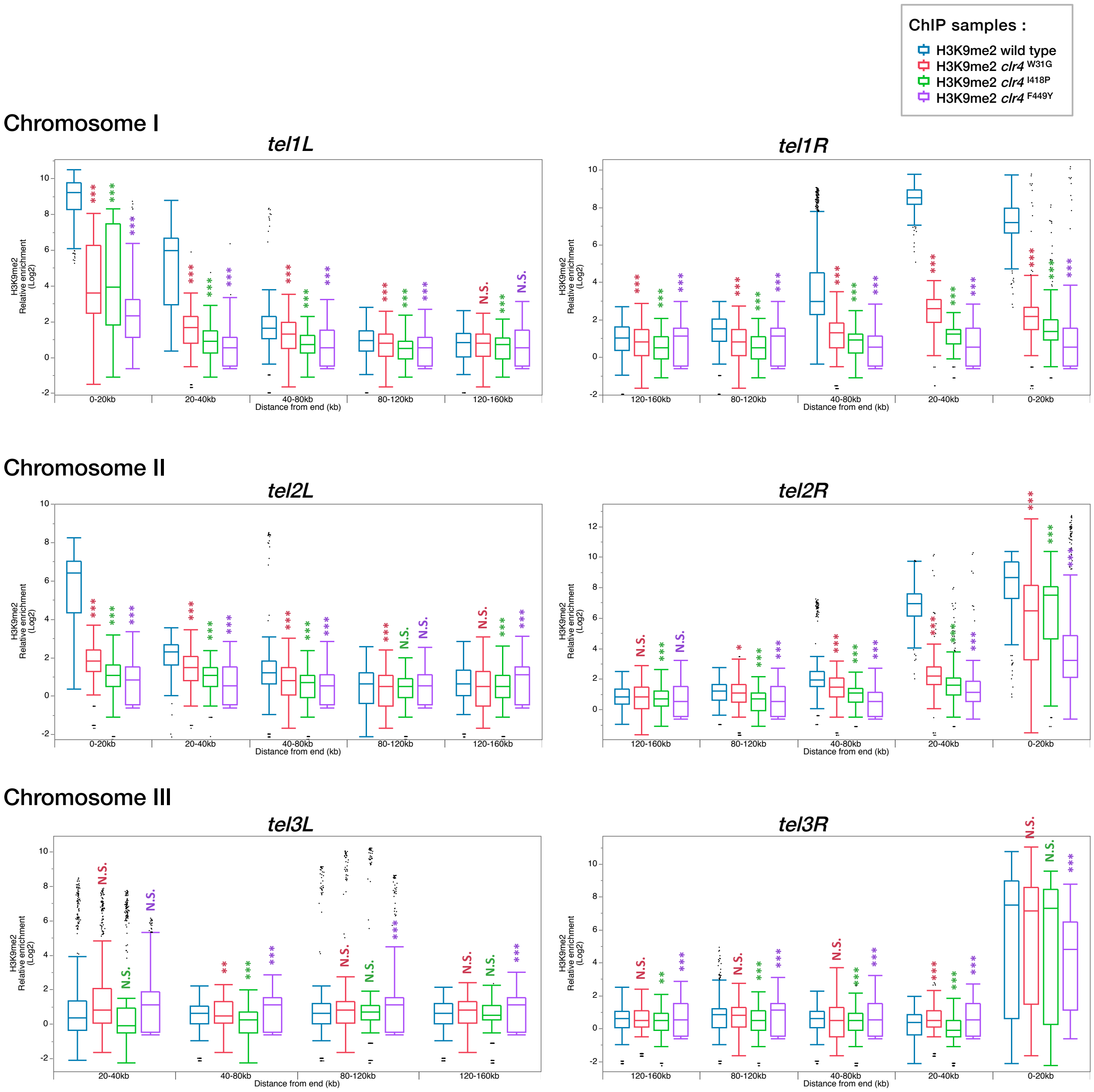

Supplementary Figure S7

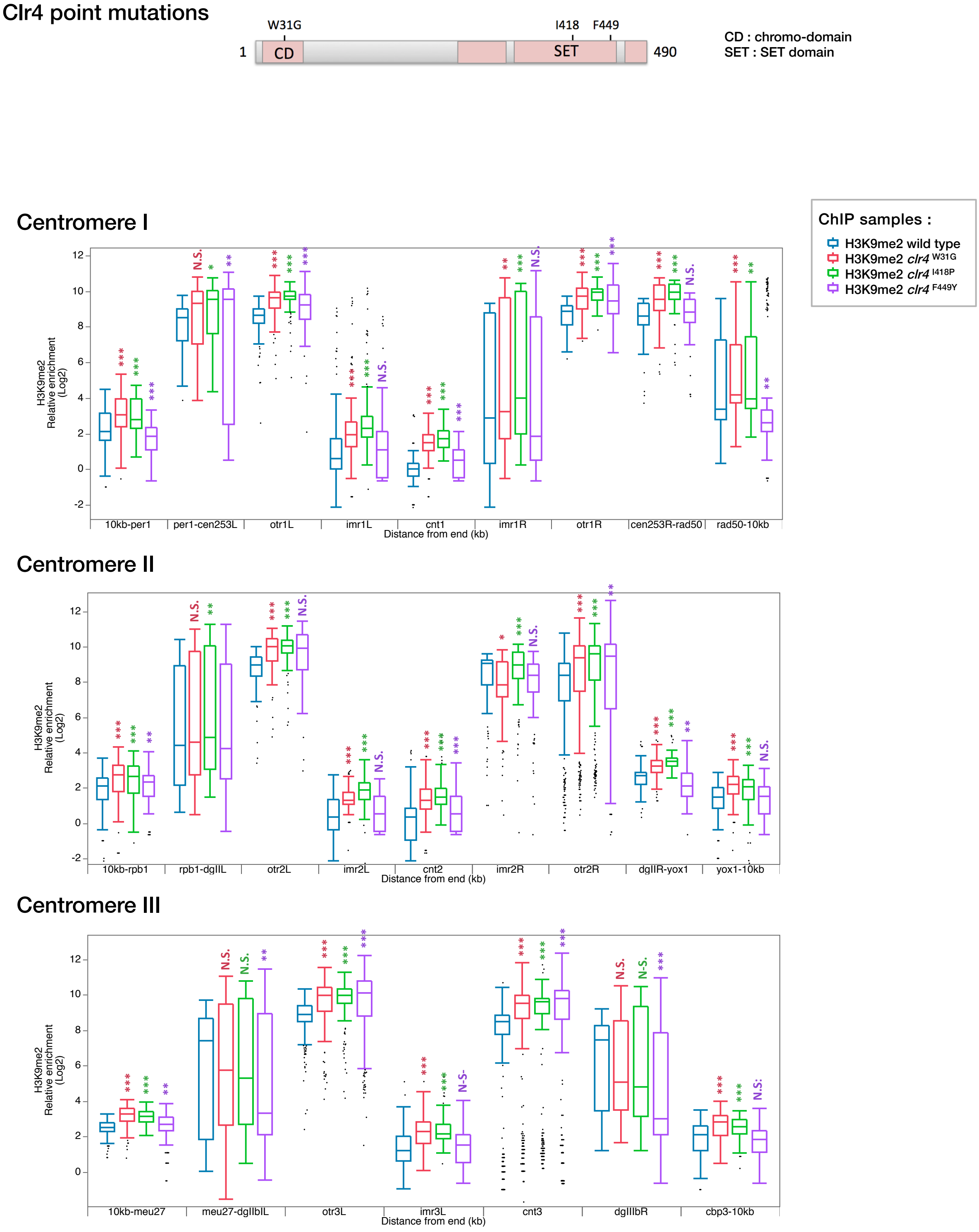

Supplementary Figure S8

A

					Zofall et al., Mol Cell, 2016		*Zofall et al. Science, 2012*					*This study*	
Island name	Island Type	Chr	Start	End	H3K9me2 wild-type (log2)	Ratio *taz1Δ*	H3K9me2 wild-type (log2)	Ratio *dcr1Δ*	Ratio *sir2Δ*	Ratio *epe1Δ*	Ratio nitrogen-starved wild-type	H3K9me2 wild-type (log2)	Ratio *abo1Δ* vs wt
Island 1	DSR	1	579066	581791	2,3	3,6	3,7	0,4	0,1	4,1	0,1	7,6	0,1
Island 2	DSR	1	2447216	2448367	1,1	1,7	1,2	0,7	0,4	1,2	0,4	1,4	0,7
Island 4	DSR	1	3647841	3650229	3,1	0,9	2,4	0,7	0,2	1,5	0,3	3,0	0,6
Island 5	DSR	1	3727551	3729743	2,0	1,1	2,8	0,2	0,2	0,6	0,8	2,4	0,5
Island 6	DSR	1	4534560	4537423	3,4	2,1	4,1	0,2	0,1	4,4	0,2	6,1	0,1
Island 8	DSR	2	899213	902307	2,5	1,5	2,5	0,5	0,2	11,9	0,2	1,8	0,5
Island 9	DSR	2	1473004	1476767	3,5	2,0	4,7	0,2	0,1	1,4	0,1	7,3	0,05
Island 16	DSR	2	3628602	3630143	1,6	1,4	1,8	0,8	0,3	2,1	0,3	1,4	0,6
Island 17	DSR	2	3641573	3642435	1,8	1,4	1,8	1,0	0,3	1,4	0,5	0,9	1,0
Island 20	DSR	3	2368972	2371127	1,7	1,5	2,0	0,3	0,3	5,9	0,2	1,5	0,7
Island 3	non-DSR	1	2522570	2523842	2,0	0,2	1,8	1,0	0,3	4,8	0,4	1,0	2,6
Island 13	non-DSR	2	1870135	1871228	1,5	0,3	1,6	0,9	0,3	1,8	0,3	1,2	1,0
Island 14	non-DSR	2	2199839	2202230	3,0	0,1	2,2	1,7	0,2	1,9	0,4	0,9	158
Island 15	non-DSR	2	2339483	2342596	2,1	0,3	2,3	0,8	0,3	1,1	0,6	5,0	6,3

B

		H3K9me2 ChIP				H3K9me3 ChIP			
Island name	Island Type	H3K9me2 WT (log2)	*Ratio clr4* ^{W31G} *vs WT*	*Ratio clr4* ^{I418P} *vs WT*	*Ratio clr4* ^{F449Y} *vs WT*	H3K9me3 WT (log2)	*Ratio clr4* ^{W31G} *vs WT*	*Ratio clr4* ^{I418P} *vs WT*	*Ratio clr4* ^{F449Y} *vs WT*
Island 1	DSR	4,8	0,2	0,1	0,1	3,0	0,6	0,6	0,4
Island 2	DSR	1,8	0,9	0,4	0,4	1,0	1,0	1,4	1,7
Island 4	DSR	2,4	0,8	0,5	0,5	3,3	0,4	1,5	1,1
Island 5	DSR	3,7	1,1	0,4	0,2	2,7	1,2	0,9	0,3
Island 6	DSR	4,9	0,1	0,0	0,0	3,1	0,1	0,7	0,5
Island 8	DSR	3,3	0,4	0,2	0,2	2,3	0,1	1,4	1,0
Island 9	DSR	5,4	0,3	0,1	0,1	3,4	0,1	0,7	0,5
Island 16	DSR	2,1	0,8	0,2	0,4	1,0	1,0	1,0	0,9
Island 17	DSR	1,3	0,9	0,4	0,6	1,6	0,3	1,4	1,0
Island 20	DSR	3,2	0,3	0,1	0,2	2,7	0,2	1,2	1,1
Island 3	non-DSR	1,5	2,1	0,8	0,6	0,6	0,7	0,9	0,6
Island 13	non-DSR	2,8	1,1	0,3	0,2	0,5	0,7	1,3	0,4
Island 14	non-DSR	3,2	0,8	0,2	0,2	2,2	0,2	0,6	0,2
Island 15	non-DSR	4,8	2,9	1,8	0,1	4,1	0,1	0,2	0,1

Supplementary Figure S9

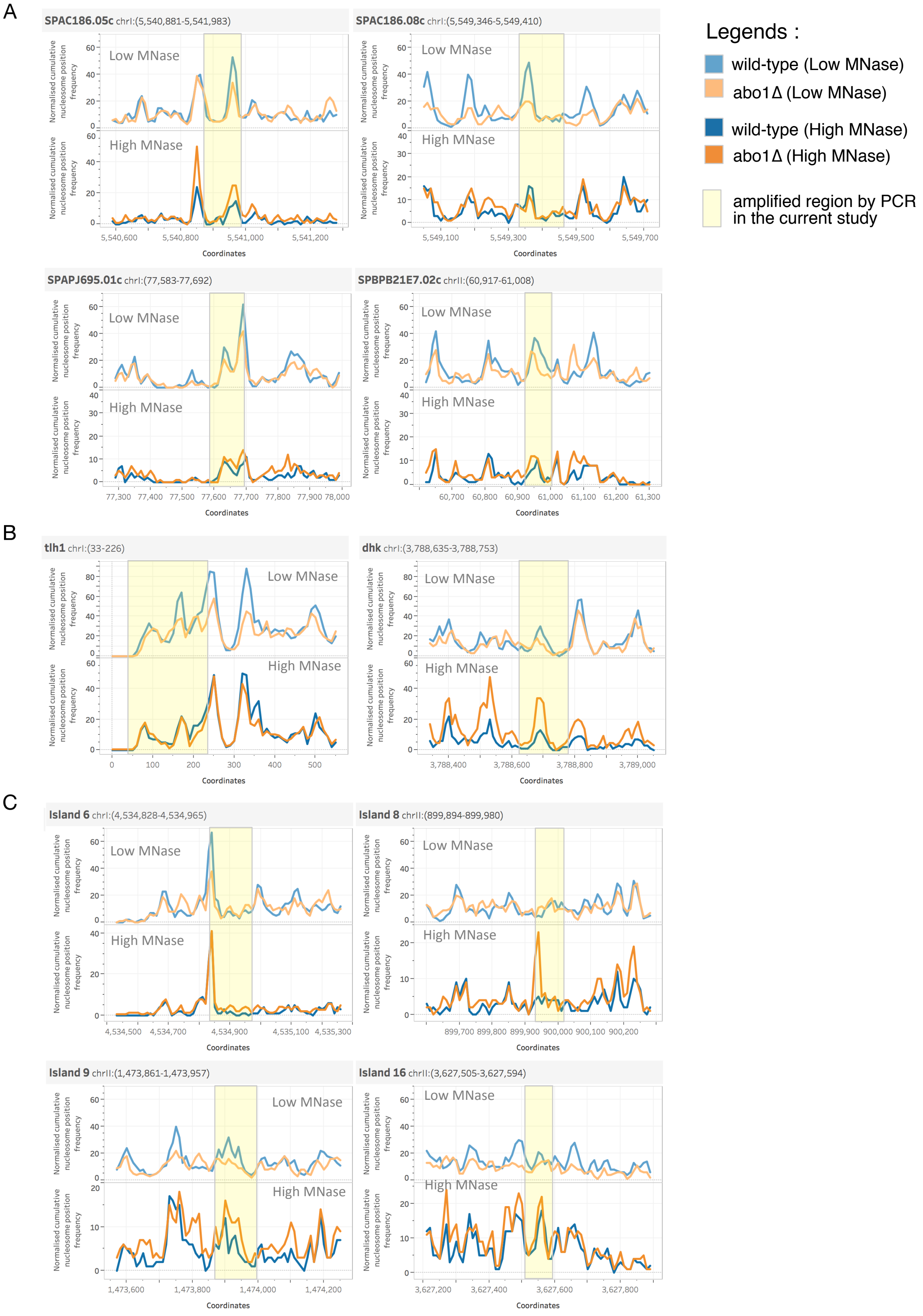

Supplementary Figure S10

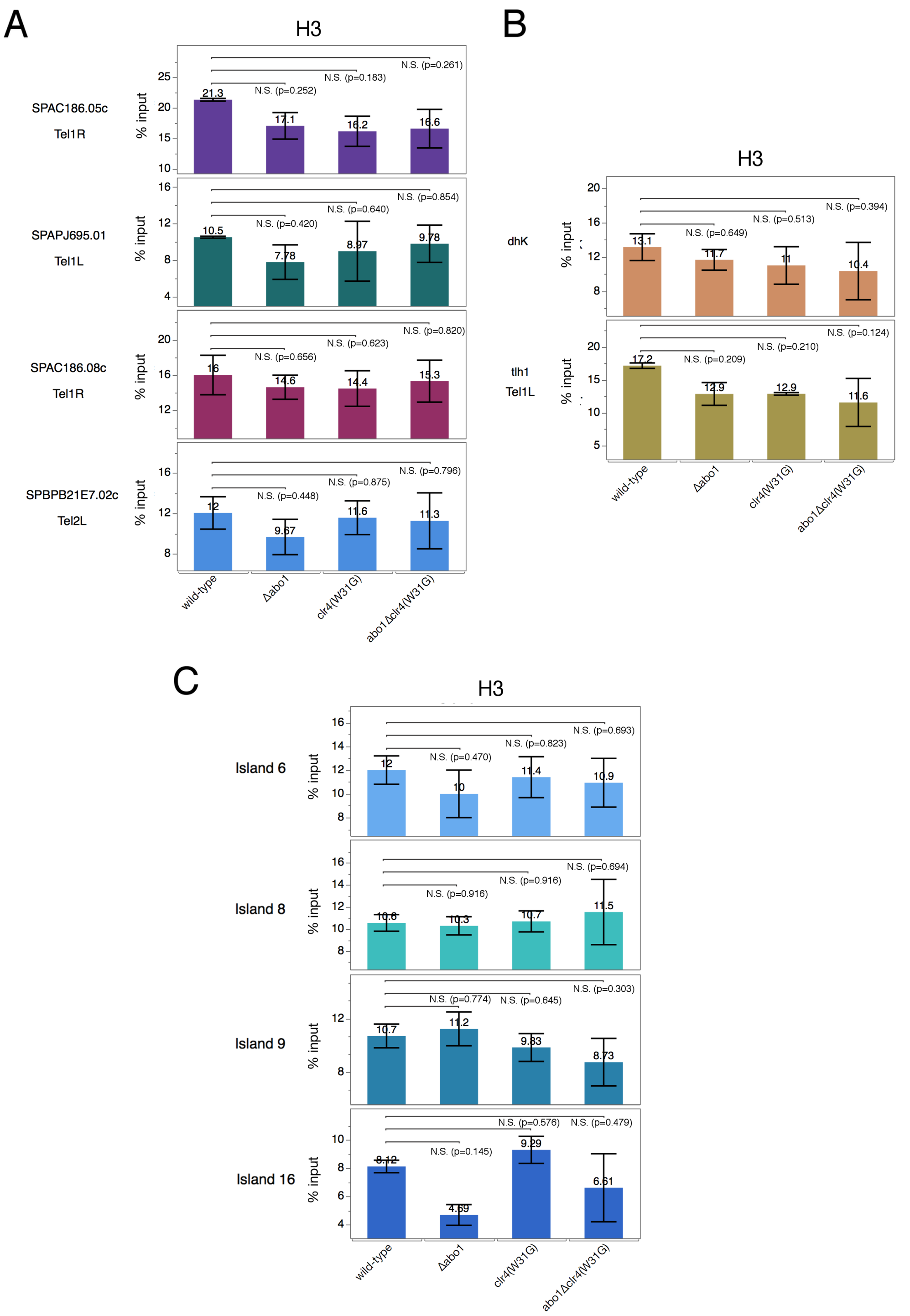

Supplementary Figure S11 (1/2)

Number of Unique Peptides		Top Identified Proteins identified only in Abo1-TAP-TAG		
**Mock**	**SPAC31G5**	**Gene ID**	**Gene name**	**Description**
2	22	SPBC2F12.05c	osh2	sterol intermembrane transfer protein Osh2 (predicted)
0	22	SPBC31E1.06	bms1	GTP binding protein Bms1 (predicted)
3	22	SPAC19D5.04	ptr1	HECT-type ubiquitin-protein ligase E3 Ptr1
0	17	SPCC16C4.08c	skb15	p21 activated protein kinase inhibitor Skb15
0	15	SPCC645.14c	sti1	chaperone activator Sti1 (predicted)
0	14	SPBC11C11.02	imp2	F-BAR domain protein Imp2
0	14	SPBC8D2.10c	rmt3	type I ribosomal protein arginine N-methyltransferase Rmt3
0	13	**SPAC31G5.19**	**abo1**	**ATPase with bromodomain protein**
2	13	SPCC18B5.01c	bfr1	plasma membrane brefeldin A efflux transporter Bfr1
2	13	**SPAC23G3.01**	rpb2	RNA polymerase II complex subunit Rpb2
0	13	SPAC4A8.12c	sds22	protein phosphatase regulatory subunit Sds22
0	13	SPCC11E10.07c	tif221	translation initiation factor eIF2B alpha subunit
0	12	SPAC343.14c	tif222	translation initiation factor eIF2B beta subunit
0	11	SPAC20G8.06	not1	CCR4-Not complex scaffold subunit Not1
2	11	**SPBC609.05**	**pob3**	**histone H2A-H2B chaperone, FACT complex subunit Pob3**
0	11	**SPBC16D10.07c**	**sir2**	**Sirtuin family histone deacetylase Sir2**
2	11	SPBC17G9.09	tif213	translation initiation factor eIF2 gamma subunit
2	10	SPBC651.11c	apm3	AP-3 adaptor complex subunit Apm3 (predicted)
**0**	**10**	**SPBC17G9.02c**	**cdc73**	**RNA polymerase II accessory factor, Cdc73 family (predicted)**
2	10	SPAC1B3.05	not3	CCR4-Not complex NOT box subunit Not3/5
0	10	SPBC428.19c	utp15	U3 snoRNP protein Utp15 (predicted)
0	10	SPAC890.04c	ytm1	ribosome biogenesis protein Ytm1 (predicted)
0	9	SPBC3F6.01c	SPBC3F6.01c	serine/threonine protein phosphatase (predicted)
0	9	SPAC4F8.12c	spp42	U5 snRNP complex subunit Spp42
0	8	SPAC1093.05	hca4	ATP-dependent RNA helicase Hca4 (predicted)
0	8	SPAC6B12.15	cpc2	G protein beta subunit-like protein Rkp1
0	7	SPAC30C2.04	asc1	cofactor for methionyl-and glutamyl-tRNA synthetases (predicted)
0	7	SPAC694.02	SPAC694.02	DEAD/DEAH box helicase
0	7	SPAC23C11.11	cka1	serine/threonine protein kinase Cka1
1	7	SPAC222.07c	hri2	eIF2 alpha kinase Hri2
0	7	SPAC664.05	rpl13	60S ribosomal protein L13
0	7	SPBC16G5.14c	rps3	40S ribosomal protein S3
0	7	**SPAPB8E5.09**	**rvb1**	**AAA family ATPase Rvb1**
1	7	SPCC188.06c	srp54	signal recognition particle subunit Srp54
0	7	SPAC22H12.02	tfg3	transcription factor TFIIF complex subunit Tfg3
0	7	SPCC24B10.21	tpi1	triosephosphate isomerase
1	7	SPBC1778.01c	zuo1	zuotin (predicted)
0	7	SPBC215.12	SPBC215.12	GTPase Cwf10 (predicted)
**0**	**6**	**SPAC16A10.02**	sub1	**transcription coactivator PC4**
0	6	SPAC977.14c	SPAC977.14c	aldo-keto reductase family protein
**0**	**6**	**SPBC13G1.08c**	ash2	**Ash2-trithorax family protein**
0	6	SPBC3D6.02	but2	But2 family protein But2
0	6	SPBC776.02c	dis2	serine/threonine protein phosphatase PP1
0	6	SPBC16E9.05	erg6	delta-sterol C-methyltransferase Erg6 (predicted)
0	6	SPAC19B12.05c	fcp1	CTD phosphatase Fcp1
1	6	SPBC36B7.09	gcn2	eIF2 alpha kinase Gcn2
0	6	SPCC1739.12	ppe1	serine/threonine protein phosphatase Ppe1
0	6	SPAC521.05	rps802	40S ribosomal protein S8
0	6	SPAC823.08c	rrp3	ATP-dependent RNA helicase Rrp3 (predicted)
0	6	SPAC57A10.10c	sla1	La-motif domain-containing protein
0	6	SPAC16E8.15	tif45	translation initiation factor eIF4E, 4F complex subunit
0	6	SPAC23H4.15	tsr1	ribosome biogenesis protein Tsr1 (predicted)
0	5	SPAC12G12.06c	rcl1	RNA 3'-terminal phosphate cyclase (predicted)
0	5	SPBC3D6.12	dip2	U3 snoRNA associted protein Dip2 (predicted)
1	5	SPAC458.02c	SPAC458.02c	mRNP complex (predicted)

Supplementary Figure S11 (2/2)

Number of Unique Peptides		Identified Proteins identified in Abo1-TAP-TAG		
Mock	SPAC31G5	Gene ID	Gene name	Description
0	5	SPCC31H12.08c	ccr4	CCR4-Not complex subunit Ccr4 (predicted)
0	5	SPCC1223.07c	drs1	cytoplasmic aspartate-tRNA ligase Drs1 (predicted)
0	5	SPCC622.09	htb1	histone H2B alpha Htb1
0	5	SPBC19G7.16	iws1	transcription elongation factor complex subunit Iws1 (predicted)
0	5	SPAC3F10.17	ltv1	ribosome biogenesis protein Ltv1 (predicted)
0	5	SPCC736.12c	mmi1	YTH family protein Mmi1
0	5	SPAC20G8.09c	nat10	N-acetyltransferase Nat10 (predicted)
0	5	SPBC14C8.14c	pol5	DNA polymerase phi
0	5	SPAC29A4.08c	prp19	ubiquitin-protein ligase E4
0	5	SPAC57A7.08	pzh1	serine/threonine protein phosphatase Pzh1
0	5	SPAC23C4.15	rpb5	DNA-directed RNA polymerase I, II and III subunit Rpb5
1	5	SPBC11C11.08	srp1	Srp1
0	5	SPCC576.13	swc5	Swr1 complex subunit Swc5
0	5	SPAC32A11.04c	tif212	translation initiation factor eIF2 beta subunit (predicted)
0	5	SPBC23E6.04c	utp10	U3 snoRNP-associated protein Utp10 (predicted)
0	4	SPBC29A10.12	oxs1	HMG-box variant
0	4	SPBC36B7.08c	ccp1	histone chaperone, CENP-A nucleosome disassembly
0	4	SPBC25B2.10	SPBC25B2.10	Usp (universal stress protein) family protein
0	4	SPCC550.15c	rei1	ribosome biogenesis protein (predicted)
0	4	SPBC1289.11	spf38	splicing factor Spf38
0	4	SPBC31F10.11c	cwf4	complexed with Cdc5 protein Cwf4
0	4	SPAC22A12.11	dak1	dihydroxyacetone kinase Dak1
0	4	SPBP8B7.16c	dbp2	ATP-dependent RNA helicase Dbp2
0	4	SPBC28E12.05	esf2	U3 snoRNP-associated protein Esf2 (predicted)
0	4	SPAC1834.03c	SPAC1834.03c	histone H4 h4.2
0	4	SPAC16E8.06c	nop12	RNA-binding protein Nop12
0	4	SPBC215.07c	pdp2	PWWP domain protein
0	4	SPBC28F2.03	ppi1	cyclophilin family peptidyl-prolyl cis-trans isomerase Cyp2
0	4	SPBC19F5.05c	ppp1	pescadillo-family BRCT domain protein
0	4	SPBC6B1.10	prp17	splicing factor Prp17
0	4	SPBC23G7.08c	rga7	Rho-type GTPase activating protein Rga7
0	4	SPAC26A3.07c	rpl1101	60S ribosomal protein L11
0	4	SPAC1805.13	rpl14	60S ribosomal protein L14 (predicted)
0	4	SPAC589.10c	ubi5	ribomal-ubiquitin fusion protein Ubi5
0	4	SPAC10F6.06	vip1	RNA-binding protein Vip1
0	3	SPBC3B8.09	utp3	U3 snoRNP-associated protein Utp3 (predicted)
0	3	SPAC1565.05	utp8	sequence orphan
0	3	SPCC1672.07	utp21	U3 snoRNP-associated protein Utp21 (predicted)
0	3	SPBC713.04c	pwp2	U3 snoRNP-associated protein Utp1
0	3	SPBC16E9.02c	SPBC16E9.02c	CUE domain protein
0	3	SPBC9B6.10	cdc37	Hsp90 co-chaperone Cdc37
0	3	SPAC644.12	cdc5	cell division control protein Cdc5
0	3	SPAC8C9.03	cgs1	cAMP-dependent protein kinase regulatory subunit Cgs1
0	3	SPBC211.02c	cwf3	complexed with Cdc5 protein Cwf3
0	3	SPAC29A4.20	elp3	elongator complex, histone acetyltransferase subunit Elp3 (predicted)
0	3	SPAC17A5.14	exo2	exonuclease II Exo2
0	3	SPAC664.01c	swi6	chromodomain protein Swi6
0	3	SPAC4F8.07c	hxk2	Hexokinase 2
0	3	SPBC1D7.04	mlo3	RNA annealing factor Mlo3
0	3	SPBC56F2.02	rpl1901	60S ribosomal protein L19
0	3	SPBC106.18	rpl2501	60S ribosomal protein L25 (predicted)
0	3	SPCC613.05c	rpl35	60S ribosomal protein L35
0	3	SPBC11C11.09c	rpl502	60S ribosomal protein L5
0	3	SPBC18H10.12c	rpl701	60S ribosomal protein L7
0	3	SPAPJ698.02c	rps002	40S ribosomal protein S0B
0	3	SPAC31G5.17c	rps1001	40S ribosomal protein S10
0	3	SPBC21C3.13	rps1901	ribosomal protein S16 homolog
0	3	SPBC17G9.07	rps2402	40S ribosomal protein S24
0	3	SPCC31H12.05c	sds21	serine/threonine protein phosphatase Sds21
0	3	SPAC23H3.05c	swd1	Set1C complex subunit Swd1
0	3	SPAC2G11.15c	tgs1	RNA methyltransferase Tgs1 (predicted)
0	3	SPAC9E9.13	wos2	p23 chaperone protein wos2

Supplementary Figure S12

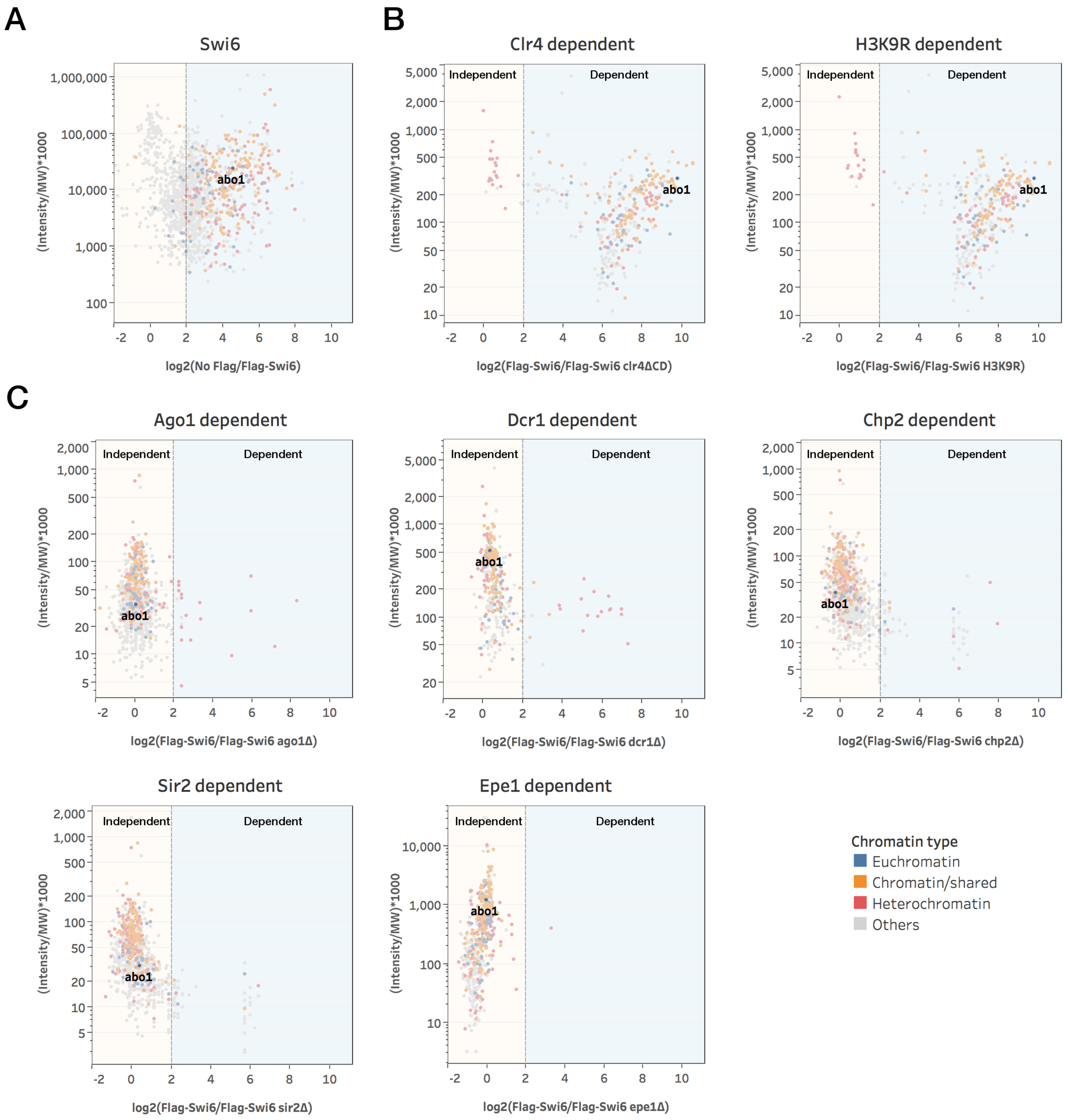

Supplement: Supplementary file 1 — Supplementary Dataset. [file 41598_2020_63209_MOESM1_ESM.pdf]
