# Supplementary material for: Abo1 is required for the H3K9me2 to H3K9me3 transition in heterochromatin
Source: Sci Rep. 2020 Apr 8;10:6055. doi: 10.1038/s41598-020-63209-y (PMC7142091; doi:10.1038/s41598-020-63209-y)
Supplement: Supplementary file 2 — Supplementary information file. [file 41598_2020_63209_MOESM2_ESM.pdf]

Supplementary information file

Abo1 is required for the H3K9me2 to H3K9me3 transition in heterochromatin

Wenbo Dong[1], Eriko Oya[1], Yasaman Zahedi[1], Punit Prasad[1,2], J Peter Svensson[1], Andreas Lennartsson[1], Karl Ekwall[1], Mickaël Durand-Dubief[1,*].

[1]Dept of Biosciences and Nutrition, Karolinska Institutet NEO, 141 83 Huddinge, Sweden

[2]Institute of Life Sciences, NALCO Square, *Bhubaneswar,* 751023 Odhisa, India

*Corresponding author :

mickael.durand-dubief@ki.se

Supplementary Table S1 The top 30 chromatin-involved genes having negative interactions *with abo1*. The score represents the strength of the negative genetic interaction. The right column represents the average standard deviation for triplicate experiments.

Supplementary Table S2 Strain table.

Supplementary Table S3 Primer table.

Supplementary Figure S1 H3K9me2, H3K9me3 enrichment and RNA expression at subtelomeric regions in the *abo1*Δ and wild-type strains. H3K9me2 enrichment (upper panel), H3K9me3 enrichment (middle panel) and expression (lower panel) in log2 at 30°C in the specified left and right subtelomere regions of all three chromosomes. P-values were calculated using a two-tailed unpaired t-test. N.S., not significant ($p>0.1$), *$p<0.1$, **$p<0.05$, ***$p<0.01$.

Supplementary Figure S2 H3K9me2 enrichment, H3K9me3 enrichment and RNA expression at different regions of the centromeres for the three chromosomes in the *abo1*Δ and wild-type strains. H3K9me2 enrichment (upper panel), H3K9me3 enrichment (middle panel) and expression (lower panel) in log2 at 30°C in the specified left and right subtelomere regions of all three chromosomes. The specified regions were labelled on the X-axis. P-values were calculated using a two-tailed unpaired t-test. N.S., not significant ($p>0.1$), *$p< 0.1$, **$p<0.05$, ***$p<0.01$.

Supplementary Figure S3 RNA expression in wild-type and abo1Δ strains at 25°C, 30°C, and 37°C. **(A)** Domainogramm with RNA expression represented as log2 values from blue (low expression) to red (high expression). **(B)** Box plots of RNA expression at different regions of the subtelomeres for the three chromosomes of the *abo1*Δ and wild-type strains. The X-axis indicates the distance from the chromosome ends. **(C)** Box plots of RNA expression at different regions of the centromeres for the three chromosomes of the *abo1*Δ and wild-type strains. The X-axis indicates the distance from the chromosome ends. P-values were calculated using a two-tailed unpaired t-test compared to the wild-type strain grown at the same temperature. N.S., not significant ($p>0.1$), *$p<0.1$, **$p<0.05$, ***$p<0.01$.

Supplementary Figure S4 Relative enrichment of H3K9me2 in wild-type, *sir2*Δ, *dcr1*Δ, *epe1*Δ, and nitrogen-starved wild-type strains at subtelomeric regions. Data were collected from previous work [1]. The X-axis represents the distance from the chromosome ends. Boxes of different colours indicate different samples as noted. P-

values were calculated using a two-tailed unpaired t-test compared to the wild-type. N.S., not significant ($p>0.1$), *$p< 0.1$, **$p<0.05$, ***$p<0.01$.

Supplementary Figure S5 Relative enrichment of H3K9me2 in wild-type and taz1Δ strains at subtelomeric regions. Data were collected from previous work [2]. The X-axis represents the distance from the chromosome ends. P-values were calculated using a two-tailed unpaired t-test relative to the wild-type. N.S., not significant ($p>0.1$), *$p<0.1$, **$p<0.05$, ***$p<0.01$.

Supplementary Figure S6 Relative enrichment of H3K9me2 at chromosome ends in wild-type, $clr4^{W31G}$, $clr4^{I418P}$, and $clr4^{F449Y}$ strains. Each strain is indicated by the noted colour. The H3K9me2 ChIP data were extracted from published data [3]. P-values were calculated using a two-tailed unpaired t-test compared to the wild-type. N.S., not significant ($p>0.1$), *$p<0.1$, **$p<0.05$, ***$p<0.01$.

Supplementary Figure S7 Relative enrichment of H3K9me2 at the centromeres of three chromosomes in wild-type, $clr4^{W31G}$, $clr4^{I418P}$ and $clr4^{F449Y}$ strains. Specified regions are labelled in the X-axis. The H3K9me2 ChIP data was extracted from published article [3]. p-values were calculated using a two-tailed unpaired t-test compared to the wild-type) N.S. (not significant) >0.1, *< 0.1; **<0.05, ***<0.01.

Supplementary Figure S8 H3K9me2 enrichment of heterochromatin islands in *abo1*Δ, *sir2*Δ, *dcr1*Δ, *epe1*Δ, and nitrogen-starved wild-type strains and H3K9me2 and H3K9me3 enrichment in $clr4^{I418P}$, $clr4^{F449Y}$, and $clr4^{W31G}$ strains. **(A)** H3K9me2 enrichment ratio in heterochromatin islands in *abo1*Δ, *sir2*Δ, *dcr1*Δ, *epe1*Δ, and nitrogen-starved wild-type strains compared to wild-type. The log2 values for H3K9me2 enrichment signals of DSR and non-DSR islands were extracted from the published data [1,2]. **(B)** H3K9me2 and H3K9me3 enrichment ratio of $clr4^{I418P}$, $clr4^{F449Y}$, and $clr4^{W31G}$ strains compared to wild-type. The log2 values for H3K9me2 enrichment signals of DSR and non-DSR islands were extracted from the published article [3] (GEO accession: GSE83495).

Supplementary Figure S9 Normalised cumulative nucleosome position frequency of wild-type and *abo1*Δ using low and high MNase of the qPCR targets. Data were obtained from Gal et al. [4] (GEO accession: GSE67410). **(A)** Subtelomeric targets. **(B)** Pericentromeric targets. **(C)** Non-DSR islands. Yellow boxes represent the regions measured by qPCR in this study. The Y-axes of the plots describe the nucleosome mid-point position frequency distributions.

Supplementary Figure S10 Histone H3 measured by ChIP-qPCR for WT, *abo1*Δ, *clr4*^{W31G}, and *abo1*Δ*clr4*^{W31G} cells. (A) H3 ChIP-qPCR occupancy of four genes located in the indicated subtelomeric regions. (B) H3 ChIP-qPCR occupancy of four genes located in pericentromeric regions and the Tel1L (tlh1) region. (C) H3 ChIP-qPCR occupancy of four different heterochromatin DSR islands: Island6 (ssm4), Island8 (mcp5), Island9 (mei4), and Island16 (pvg4). All qPCR experiments were performed in at least two independent biological experiments. Error bars indicate standard error of the mean. *p< 0.1, **p<0.05, ***p<0.001, two-tailed unpaired t-test. N.S., not significant (i.e. p>0.1).

Supplementary Figure S11 Proteins identified in Abo1-TAP. Wild-type (Mock) and abo1-TAP extracts were purified and analysed using mass spectrometry. The left column indicates the number of unique peptides detected.

Supplementary Figure S12 Proteins recovered from Flag-Swi6 nChIP purification in wild-type compared to no-FLAG background or Flag-Swi6 in a mutant background. Data were extracted from Iglesias et al. [5]. Y-axis represents the protein abundance (total peptide intensity divided by molecular weight [MW] multiplied by a 1,000) in logarithmic scale. X-axis represents the log2 ratio value of Flag-Swi6 wild-type versus no Flag or Flag-Swi6 mutation background IP. Major FLAG-Swi6 pulled-down protein groups relevant are color coded using the criteria defined in the same study [5] and abo1 protein name is denoted. Vertical dashed black lines mark an enrichment of 4-fold compared to the reference.

References :

1. Zofall M, Yamanaka S, Reyes-Turcu FE, Zhang K, Rubin C, Grewal SIS (2012) RNA Elimination Machinery Targeting Meiotic mRNAs Promotes Facultative Heterochromatin Formation. *Science* **335:** 96-100.

2. Zofall M, Smith DR, Mizuguchi T, Dhakshnamoorthy J, Grewal SIS (2016) Taz1-Shelterin Promotes Facultative Heterochromatin Assembly at Chromosome-Internal Sites Containing Late Replication Origins. *Mol Cell* **62:** 862-874.

3. Jih G, Iglesias N, Currie MA, Bhanu NV, Paulo JA, Gygi SP, Garcia BA, Moazed D (2017) Unique roles for histone H3K9me states in RNAi and heritable silencing of transcription. *Nature* **547:** 463.

4. Gal C, Murton HE, Subramanian L, Whale AJ, Moore KM, Paszkiewicz K, Codlin S, Bähler J, Creamer KM, Partridge JF, Allshire RC, Kent NA, Whitehall SK (2016) Abo1, a conserved bromodomain AAA-ATPase, maintains global nucleosome occupancy and organisation. *Embo Rep* **17**, 79-93.

5. Iglesias N, Paulo JA, Tatarakis A,Wang X, Edwards AL, Bhanu NV, Garcia BA, Haas W, Gygi SP, Moazed D. (2019) Native Chromatin ProteomicsReveals a Role for Specific Nucleoporins in Heterochromatin Organization and Maintenance. *Mol Cell* **77** 1–16,
